# ALICE: a hybrid AI paradigm with enhanced connectivity and cybersecurity for a serendipitous encounter with circulating hybrid cells

**DOI:** 10.7150/thno.44053

**Published:** 2020-09-02

**Authors:** Kok Suen Cheng, Rongbin Pan, Huaping Pan, Binglin Li, Stephene Shadrack Meena, Huan Xing, Ying Jing Ng, Kaili Qin, Xuan Liao, Benson Kiprono Kosgei, Zhipeng Wang, Ray P.S. Han

**Affiliations:** 1 College of Engineering, Peking University, Beijing 100871, China.; 2 Jiangzhong Cancer Research Center, Jiangxi University of Traditional Chinese Medicine, Nanchang, Jiangxi, China 330004.

**Keywords:** ALICE, cell phenotyping software, hybrid artificial intelligence, image forgery detection, circulating hybrid cells

## Abstract

A fully automated and accurate assay of rare cell phenotypes in densely-packed fluorescently-labeled liquid biopsy images remains elusive.

**Methods:** Employing a hybrid artificial intelligence (AI) paradigm that combines traditional rule-based morphological manipulations with modern statistical machine learning, we deployed a next generation software, ALICE (Automated Liquid Biopsy Cell Enumerator) to identify and enumerate minute amounts of tumor cell phenotypes bestrewed in massive populations of leukocytes. As a code designed for futurity, ALICE is armed with internet of things (IOT) connectivity to promote pedagogy and continuing education and also, an advanced cybersecurity system to safeguard against digital attacks from malicious data tampering.

**Results:** By combining robust principal component analysis, random forest classifier and cubic support vector machine, ALICE was able to detect synthetic, anomalous and tampered input images with an average recall and precision of 0.840 and 0.752, respectively. In terms of phenotyping enumeration, ALICE was able to enumerate various circulating tumor cell (CTC) phenotypes with a reliability ranging from 0.725 (substantial agreement) to 0.961 (almost perfect) as compared to human analysts. Further, two subpopulations of circulating hybrid cells (CHCs) were serendipitously discovered and labeled as CHC-1 (DAPI+/CD45+/E-cadherin+/vimentin-) and CHC-2 (DAPI+ /CD45+/E-cadherin+/vimentin+) in the peripheral blood of pancreatic cancer patients. CHC-1 was found to correlate with nodal staging and was able to classify lymph node metastasis with a sensitivity of 0.615 (95% CI: 0.374-0.898) and specificity of 1.000 (95% CI: 1.000-1.000).

**Conclusion:** This study presented a machine-learning-augmented rule-based hybrid AI algorithm with enhanced cybersecurity and connectivity for the automatic and flexibly-adapting enumeration of cellular liquid biopsies. ALICE has the potential to be used in a clinical setting for an accurate and reliable enumeration of CTC phenotypes.

## Introduction

Liquid biopsy in cancer research constitutes a minimally invasive procedure that can be readily carried out with relative ease 1 for sampling one of the most investigated biological materials in body fluids: circulating tumor cells (CTCs) if the body fluid is blood and mobile tumor cells (MTCs) if the body fluid is non-blood 2. The prevalence and pervasiveness of these rare cancer cells have been demonstrated to correlate well with clinical predictions for diagnosis 3, 4, prognosis 5, 6, relapse monitoring 7, 8 and treatment response 9, 10. However, the adoption of CTCs/MTCs in routine cancer management is still not widespread despite the reported efficacy of its use 11. To-date, the CellSearch system 12 remains the only FDA approved CTC-based blood test even though CTCs have been investigated for over a century 13.

One of the major hurdles impeding the acceptance of CTCs/MTCs in clinical oncology is the accuracy and reliability of the CTC/MTC count assessment 14. The current de facto standard for the identification and enumeration of these tumor cells by trained human examiners employs an immunofluorescent staining approach that involves cancer-specific markers such as the epithelial cell adhesion molecule (EpCAM) and cytokeratin (CK) 15. Inevitably, the count results are affected by human-introduced variabilities that manifest in two forms: inter-observer variability and intra-observer variability. The former is due to a subjective interpretation of the fluorescently labeled cells with disparate criteria and the latter can be attributed to the viewing of images with different hardware, as well as, fatigue arising from the highly intense labor and time involved in a typical manual operation. These observer variabilities compromise the accuracy and reliability of the CTC/MTC enumeration. Other factors that can significantly affect the accuracy and reliability of the prevalence assessment of tumor cells in body fluids are issues pertaining to CTC/MTC false positives, capture purity and cellular phenotyping. The CTC/MTC false positives occur when white blood cells (WBCs) are unintentionally labeled as tumor cells (as per the immunostaining results). The issue of capture purity refers to the inadvertent harvesting of WBCs, the amount of which is usually disproportionately larger than CTCs/MTCs. Tumor heterogeneities and cells with distinct biological functions and responses often demand that cellular phenotypic count instead of the total count be used. All these issues can forestall the establishment of liquid biopsy standards for guiding the diagnosis, staging, treatment and relapse monitoring of cancer patients.

The first step in achieving consistent and high-fidelity standards so that cellular liquid biopsy can be developed into a mainstream tool for cancer management is to replace the complexity of the challenging human enumeration assessment with a computer-aided diagnosis (CAD) system. Stott et al. 16 presented an automated imaging system for the analysis of prostate CTCs, following enrichment by the CTC-Chip 17. Their rule-based system exploited the size and shape of cells and the colocalization of fluorescent signals of the nucleus marker 4',6-diamidino-2-phenylindole (DAPI) and prostate-specific antigen (PSA) for the CTC identification. In addition to rule-based methods, advanced techniques such as statistical machine learning algorithms, e.g. the random forest 18, support vector machine (SVM) 19, and naive Bayesian classifier 20 have been used for automated detection of fluorescently stained CTCs. Furthermore, convolutional neural networks have been successfully employed to identify CTCs in dark field microscopic images of unstained blood 21. Functional software based on these computational techniques has also been developed. The *Precise and Automatic CTC Enumeration* (PACE) chip system 14 combines a specially designed microfluidic chip with an image processing algorithm to achieve an automated CTC count; however, it outputs only the CK19 positive CTCs, which implies that it can only generate the epithelial CTC count. The *Automated CTC Classification, Enumeration and PhenoTyping* (ACCEPT) software was developed under the European Union funded CANCER-ID & CTCTrap programs 22, 23 and it utilizes a deep learning algorithm for an automated CTC classification via an epithelial marker staining. Although the immunofluorescent identification of tumor cells is considered more reliable than the traditional hematoxylin and eosin (H&E) staining, software such as the CTC AutoDetect 1.0 system 24 have been developed to detect H&E stained CTCs based on morphological criteria (cell diameter > 24 µm, a non-normal oval/circular shape, etc.). This software has one major limitation - they are designed to enumerate the most common epithelial CTCs without considering other phenotypes. To the best of our knowledge, we are not aware of major software that can handle CTCs/MTCs beyond the epithelial phenotypes.

**Figure 1:**
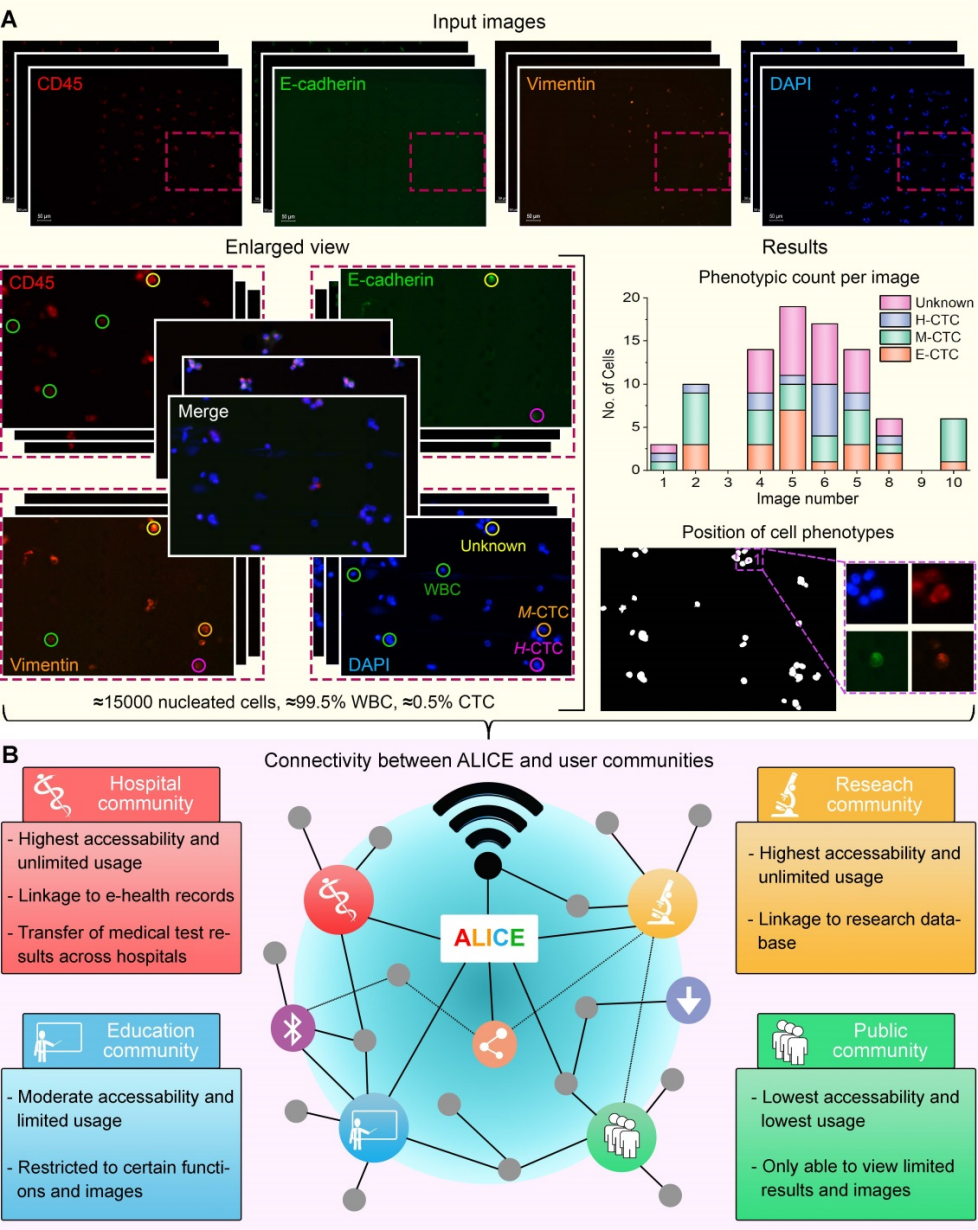
** Major operational challenges of a modern biomedical software for futurity. (A)** Rare tumor cells bestrewed in dense and massive populations of non-tumor cells require accurate processing. 'E-CTC' denotes epithelial circulating tumor cell that expressed positive for the nucleus marker DAPI and epithelial tumor marker E-cadherin but negative for the mesenchymal tumor marker vimentin and leukocyte marker CD45. 'M-CTC' denotes mesenchymal CTC that expressed positive for DAPI and vimentin but negative for E-cadherin and CD45. 'H-CTC' denotes hybrid CTC that expressed positive for DAPI, E-cadherin and vimentin but negative for CD45. 'Unknown' denotes cell that expressed positive for all 4 markers. White blood cell (WBC) expressed positive for DAPI and CD45 but negative for E-cadherin. **(B)** Enhanced software connectivity to encourage participation from appurtenant user communities. Different communities will have different accessibility and functions.

We present the software ALICE for an automated and accurate identification-cum-enumeration of multiple cellular phenotypes (up to 20) in fluorescent microscopy images. Further, for an in-depth scrutiny of the liquid biopsy data, the software can be configured to output positions and (optional) thumbnails of rare tumor cells (< 0.5%) bestrewed in dense and massive populations of WBCs (**Figure 1A**). A hybrid artificial intelligence (AI) paradigm that integrates traditional rule-based morphological manipulations with modern statistical machine learning is programmed into ALICE to manage varying cell phenotyping activities obtained from conventional and non-conventional biomarker combinations. To encourage participation from appurtenant user communities, ALICE is designed to be accessed by the following four groups: hospital, research, education and public, each with its own defined degree of access permission and usage functions (**Figure 1B**). An enhanced cybersecurity system to combat intrusive hackings and safeguard against image manipulations is built into ALICE. We benchmarked and validated the performance of ALICE using publicly reposited images sets, as well as, fluorescent image sets containing CTC phenotypes. We also described the detection of a new circulating hybrid cell population in the peripheral blood of pancreatic cancer patients. As reported here, this serendipitous discovery made using ALICE constitutes a preliminary investigation of a new fusion hybrid that appears to exhibit promising biological significance in relation to the disease progression.

## Results

### ALICE workflow and improved gray image binarization

**Figure 2:**
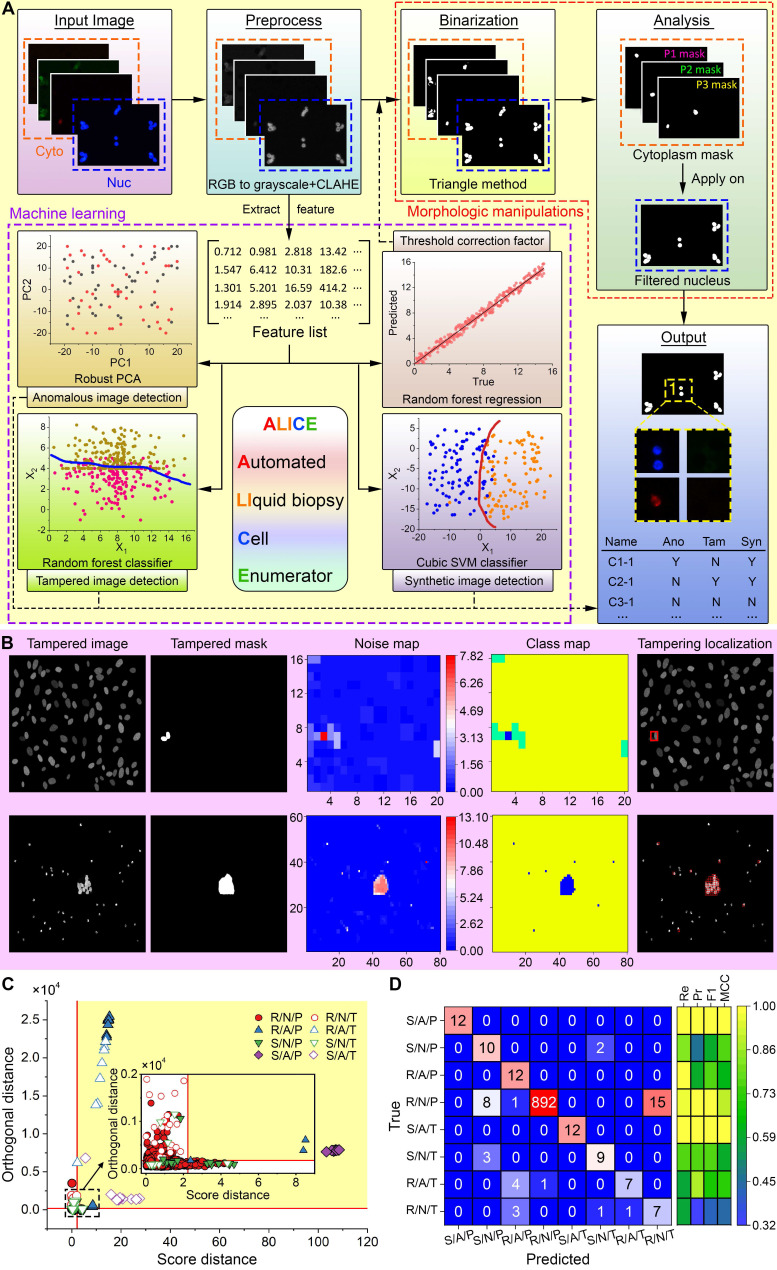
** Overview of ALICE with enhanced connectivity and cybersecurity. (A)** ALICE contains a hybrid AI engine that combines traditional rule-based morphological manipulations with modern statistical machine learning for an automated and accurate identification, localization and enumeration of cell phenotypes. The built-in cybersecurity detects enigmatic and fake input data. **(B)** Localization of the tampered region via a multi-class hierarchical clustering based on PCA-derived noise levels of the input image. Different colors in the class map represent different predicted classes. The class with the least number of pixels is considered a tampered region and visualized by a bounded red box. **(C)** From an image set of 1000 images, individually detected image types are marked using different symbols. The yellow shaded area represents the region of anomaly determined by the robust PCA. Inset shows a magnified plot of the region around the origin. Anomalous, tampered and synthetic images are detected by the robust PCA algorithm, random forest classifier and cubic SVM classifier, respectively. Note: 'R' denotes real, 'S' denotes synthetic, 'N' denotes normal, 'P' denotes pristine, 'A' denotes anomalous, 'T' denotes tampered. **(D)** The corresponding confusion matrix and performance indices for the detection results in **(C)**. 'Re' denotes the recall, 'Pr' denotes the precision, 'F1' denotes the F1 score and 'MCC' denotes the Matthews correlation coefficient.

ALICE workflow consists of five major steps (**Figure 2A**); the 1st is a preprocessing of input images into grayscale data (for RGB input), followed by a two-part 2nd step: data denoising via a bilateral filtering technique 25 and contrast enhancing via a contrast-limited adaptive histogram equalization (CLAHE) algorithm 26. The third step involves a binarization of the data into black (background) and white (foreground) pixels. The Triangle technique 27 is then used to generate superior gray image thresholding results, which were compared to the results of 15 other thresholding methods in **Figure S1** (see Methods for the details of the thresholding method). To flexibly and accurately binarize images under varying ambient conditions (e.g. lighting, contrast level, etc.), a thresholding correction factor is introduced to modulate the Triangle thresholding value. The final thresholding value is a product of the initial value and the correction factor. To estimate the thresholding correction factor, we employed 14 regression machine learning models to automatically select a parametric value that is concordant with the image's features ('Binarization' in **Figure 2A**). Working with the 10×10-fold cross-validation and test sets, we found that the ensemble method model with the random forest kernel function yielded the least test set error (root mean square error=0.74 (95% CI: 0.70-0.81) and the mean absolute error=0.50 (95% CI: 0.47-0.52) (**Table S1**)). The Watershed algorithm 28 is used to separate overlapping and clustered cells. The 4th step involves creating cytoplasmic masks and purging non-nucleic objects in the nucleus channel image via traditional morphologic criteria of cell size and eccentricity. The cytoplasmic masks are applied to the filtered nucleus image and a rule-based algorithm is invoked to identify, locate and enumerate the different cell phenotypes ('Analysis' in **Figure 2A**). The 5th step pertains to a data output of the cellular information - the enumeration of each cell phenotype and the corresponding location of each identified cell. Additionally, thumbnails of the cell phenotype can be exported for downstream confirmation and analysis. ALICE currently supports up to one nucleus channel and three cytoplasmic channels for a total of twenty cell combinations/phenotypes (**Table S2**). A graphical user interface (GUI) for pellucid point-and-click operations is used to specify the number of channels for a field of view (FOV) with images ordered to the bright field, nucleus or the 3 cytoplasm markers (**Figure S2**).

### Internet of things (IOT) connectivity and enhanced cybersecurity for enigmatic and fake images

As an advanced software designed for futurity, ALICE serves not only as a tool for biomedical research but also, as a databank for academic teaching and continuing education for extended user communities. This open-source accessibility via a cloud-based internet of things (IOT) connectivity requires enhanced cybersecurity against digital attacks that are aimed at accessing, planting, modifying or destroying sensitive data. ALICE engages several advanced tools such as the robust principal component analysis (PCA) algorithm, random forest classifier and cubic SVM classifier to identify and flag enigmatic and fake images ('Machine Learning' box in **Figure 2A**). The software adopts the same extracted features used in the determination of the threshold correction factor to implement one unsupervised classification model and train 2 separate single-target classification models for the detection of anomalous, tampered and synthetic images, respectively (**Figures 2B-D**). Further, we used a single-target approach instead of the multi-label method 29 to handle all three types of manipulated images. This approach allows for an easy treatment of the three image types together with any of their combinations via a quad-layered protection system.

The first protective layer is to curb anomalous images using the robust PCA algorithm 30 and we do this by tuning and optimizing two parameters: *k*, the number of principal components to retain and *α*, the lower bound of the proportion of uncontaminated observation. Experimenting with varying amounts of anomalies, we found the optimal parameters to be *k* = 1, *α* = 0.70 (**Table S3**). The second layer of protection deals with the detection of tampered images and forgeries. With the easy availability of advanced image editing tools, it has become increasingly challenging to detect manipulated images as they are virtually indistinguishable from real images. We trained and tested 22 classifiers using a large image set obtained by automatically splicing and pasting up to three objects randomly in the ground truth mask of publicly available image sets 31. This procedure generated 46,936 images (50% pristine-tampered pairs) and these images were segregated into 37,549 (80%) training images and 9,387 (20%) testing images. Among the 22 classification models for detecting tampered and forged images, the random forest approach produced the best performance with a test sensitivity = 97.38% (95% CI: 95.82-97.79%), specificity = 97.78% (95% CI: 97.33-98.20%) and accuracy = 97.58% (95% CI: 97.22-97.88%) (**Table S4**). The third protective layer involves the detection of synthetic images using a different classification model from the one employed for tampering detection. Trained and tested on 12,448 (80:20 ratio for train:test) real and synthetic fluorescent images, we found four classifiers: logistic regression, quadratic SVM, cubic SVM and random forest classifiers all performed equally best among the 22 models (**Table S5**). The fourth layer of protection is the localization of image tampering which, in most cases, is indiscernible to the naked eye. To achieve this localization, we adapted the multi-class hierarchal clustering technique 32 to work with the PCA-derived noise of the image 33 and assumed that the class with the least number of pixels is the tampered region. **Figure 2B** depicts two examples of tampering localization; the top row illustrates a small tampered region (0.3% tampered pixels) in a synthetic/normal/tampered image and it is visually indistinguishable from the main image, and the bottom row shows a larger tampered region (1.3% tampered pixels) in a synthetic/anomalous/tampered image. For both cases, ALICE was able to accurately localize the tampered region despite generating some small misidentified patches in the second example.

The final test is to evaluate the performance of ALICE in a realistic image set where anomalous, tampered and synthetic images often appeared as integrated together. For this purpose, we curated a new image set of 1000 real (R), synthetic (S), normal (N), pristine (P), anomalous (A), and tampered (T) images distributed as follows: 916 R/N/P and 12 of each R/N/T, R/A/P, R/A/T, S/N/P, S/N/T, S/A/P, S/A/T. **Figures 2C-D** show the final result in the detection of combined anomalous, tampered and synthetic images. When dealing with more obvious abnormalities such as S/A/P, S/N/P, R/A/P, R/N/P and S/A/T image types, the cubic SVM classifier in ALICE was able to detect almost all with a high recall, precision, F1 score and Matthews correlation coefficient (MCC) (**Figure 2D**). However, when detecting the more difficult abnormalities (S/N/T, R/A/T and R/N/T) ALICE's performance decreased somewhat despite having correctly classified a majority of these image types (**Figure 2D** with details in **Table S6**).

### Performance assessment in synthetic and real fluorescent images

**Figure 3:**
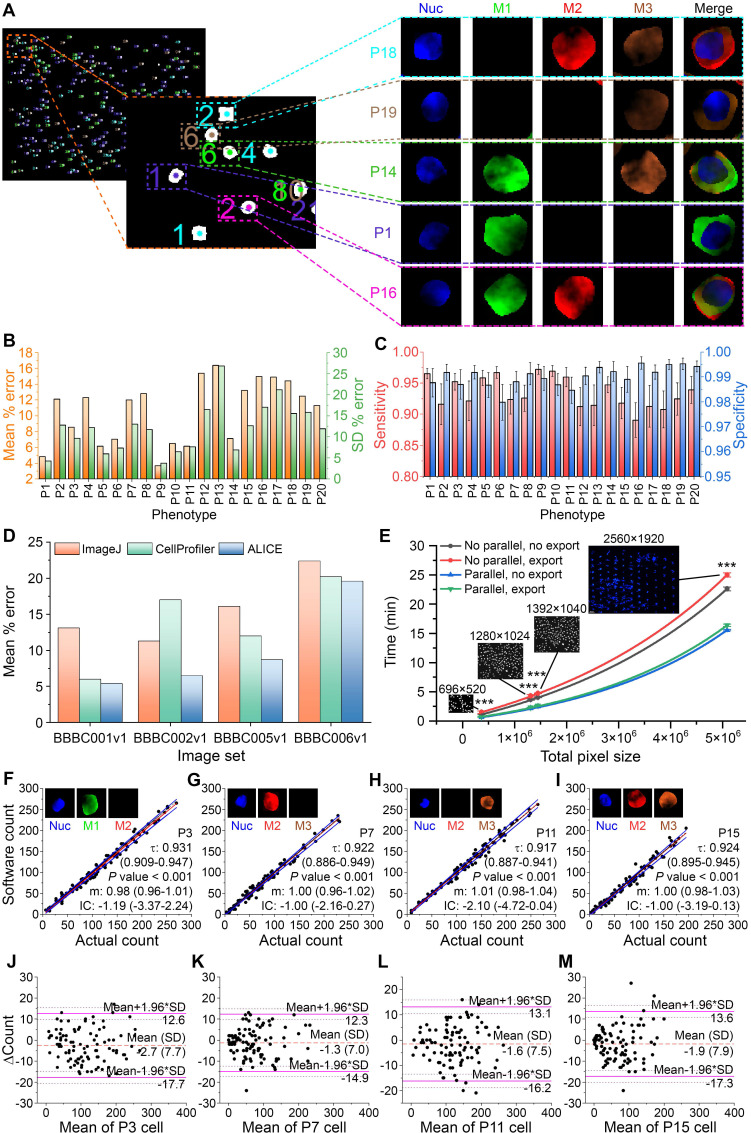
** Assessing ALICE's performance and capabilities. (A)** Identification, localization and enumeration of 5 representative simulated cell phenotypes among 300 synthetic cells in a sample image along with output cell thumbnails for 4 channels: Nuc, M1, M2 and M3 that denote respectively, the nucleus and cytoplasm marker 1, 2, 3. **(B)** Mean percentage error and SD in the enumeration of 20 cell phenotypes (all *n*=100). **(C)** Sensitivity and specificity in the enumeration of 20 cell phenotypes (all *n*=100). Error bars represent the 95% CI. **(D)** Assessing ALICE cell enumerative performance against ImageJ and CellProfiler using 4 publicly available real image sets. **(E)** Characterizing the processing time with respect to image size, processing scheme and result export. Insets show representative image and size dimension. Error bars represent SD. *** - *P* < 0.001. **(F-I)** Passing-Bablok regression analysis for P3, P7, P11 and P15 (all *n*=100) with results of the remaining phenotypes shown in Figure S3. Black dash lines represent the identity line, red solid lines represent the fitted line and blue solid lines represent the 95% CI of the fitted line. Insets depict individual fluorescent channel images, τ denotes the Kendall's correlation coefficient and m denotes the slope. **(J-M)** Bland-Altman plots for the same 4 phenotypes (all *n*=100) with results of the remaining phenotypes shown in Figure S4. ΔCount denotes the difference between the 2 counts. Orange dash lines represent the mean difference between ALICE's count and the simulated ground truth, purple solid lines represent the 95% limits of agreement and brown dotted lines represent the 95% CI of the limits of agreements. The phenotypes are defined in Table S2.

ALICE is designed to simultaneously enumerate multiple cell phenotypes in an image set. To handle this, the cells are color-coded for each phenotypic group and each cell within the same group is labeled with individually color-synchronized count data to serve as a unique identifier for the cell (**Figure 3A**). A synthetic fluorescent image set was employed to assess ALICE's phenotypic enumeration accuracy. It was created using SimuCell 34 and contained 2000 fluorescent images, each with 5 channels: bright field, nucleus marker, cytoplasm markers 1, 2 and 3 to generate 20 different phenotypes (**Table S2**). The assessment scheme proposed by the Broad Institute, Cambridge, MA was used for the evaluation and ALICE achieved a pooled mean percentage error ± SD of 10.6 ± 13.2% for all 20 phenotypes (**Figure 3B**). The pooled sensitivity and specificity for these phenotypes were 0.934 (95% CI: 0.801-1.000) and 0.990 (95% CI: 0.924-1.000), respectively (**Figure 3C**).

We also assessed the performance of ALICE on real fluorescent image sets by comparing against two highly popular state-of-the-art bioimage software: ImageJ 35, 36 and CellProfiler 37, 38. Unlike ALICE, both ImageJ and CellProfiler are unable to carry out a simultaneous enumeration of multiple cell phenotypes and therefore, we compared the total cell count predicted by all three software. To achieve the comparison, we selected 4 publicly available real image sets sourced from the Broad Bioimage Benchmark Collection (BBBC) 39: 1) Human HT29 Colon Cancer Cells image set (BBBC001v1); 2) Drosophila Kc167 Cells image set (BBBC002v1); 3) Simulated High-content Screening image set (BBBC005v1) and 4) Human U2OS Cells image set (BBBC006v1). As shown in **Figure 3D**, the performance of ALICE was found to be superior to either ImageJ or CellProfiler as it generated the least mean percentage error for all four sets and the difference was most striking for the BBBC002v1 image set with ALICE mean percentage error at 6.5%, which was nearly half of ImageJ (11.3%) and CellProfiler (17%).

Since image processing requires very intense computational effort, it is important to assess the software processing time. For each image size, we evaluated the processing time for the following four situations: with or without parallel processing and with or without exporting the cell thumbnails; designated, respectively as, “no parallel, no export”; “no parallel, export”; “parallel, no export”; and “parallel, export”. The processing times for 20 FOVs with image dimensions of 696×520 pixels (no parallel, no export) and 2560×1920 pixels (no parallel, export) were 1.1 min and 25.0 min, respectively. It appeared that the processing time increased exponentially with an increasing image size (adjusted *R^2^* for an exponential fitting of the four conditions are 0.999, 0.999, 0.998, 0.999) (**Figure 3E**). As expected, invoking parallel processing will significantly reduce the processing time for all image sizes (all *P* < 0.05). Also, when an image size is small (e.g. 696×520 pixels), exporting cell thumbnails did not generate a significant increase in the processing time, regardless of with or without parallel processing (*P* = 0.099 and *P* = 0.174, respectively). The converse is true for handling a large image size (e.g. 1280×1024 pixels) - exporting results caused a statistically significant increase in the processing time regardless of with or without parallel processing (all *P* < 0.05, **Figure 3E**).

Another performance metric considered is the reliability of ALICE. This was tested via an agreement analysis between ALICE's phenotypic count and the simulated ground truth using the Passing-Bablok regression method 40 (**Figure 3F-I** and details in **Figure S3**). With the assumptions of high correlation and linearity satisfied, none of the 20 phenotypes exhibited proportional bias (95% CI of the fitted slope not including 1) nor constant bias (95% CI of the fitted intercept not including 0). Further, the Bland-Altman plots 41 confirmed that no proportional bias was present for all 20 phenotypes (**Figure 3J-M** and details in **Figure S4**) but a mean bias ranging from -4.9 to 0.9 was observed. Both tests confirmed the reliability of ALICE in that it performed consistently well under a wide range of CTC counts (i.e. no proportional bias) and the deviation from the true count was small (i.e. small constant bias).

### Benchmarking ALICE CTC phenotyping enumeration against human count

**Figure 4:**
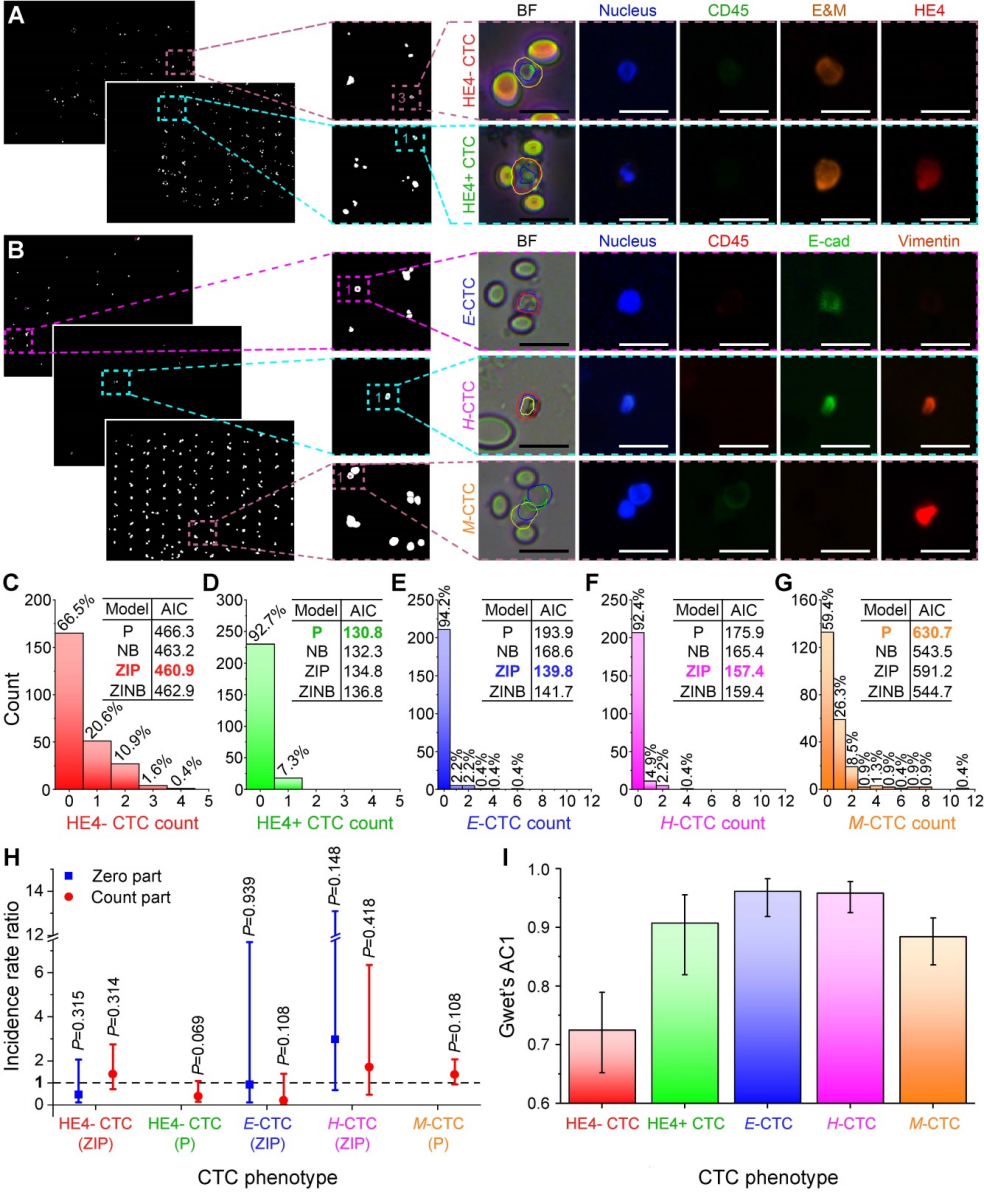
** Benchmarking ALICE CTC phenotypic count against human enumeration in real fluorescent images.** Identification, localization and enumeration of CTC phenotypes: **(A)** HE4- (DAPI+/CD45-/E&M+/HE4-) and HE4+ (DAPI+/CD45-/E&M+/HE4+) CTCs from 61 ovarian cancer patients, **(B)** *E* CTCs (DAPI+/CD45-/E-cadherin+/vimentin-), *H* CTCs (DAPI+/CD45-/E-cadherin+/vimentin+) and *M* CTCs (DAPI+/CD45-/E-cadherin-/vimentin+) from 46 pancreatic cancer patients. E&M denotes combined epithelial and mesenchymal markers. Scale bar: 20 µm. **(C-G)** Distribution of the phenotypic count for HE4- CTC, HE4+ CTC, *E*-CTC, *H*-CTC and *M*-CTC. Inset tables show the AIC values for the 4 fitted regression models: Poisson (P), negative binomial (NB), zero-inflated Poisson (ZIP) and zero-inflated negative binomial (ZINB) model and the model with the lowest AIC value is bolded and colored. **(H)** Incidence rate ratio (IRR) plot indicating the CTC phenotypic counts of ALICE and human are statistically indifferent. The fitted regression models are listed for each CTC phenotypes and the zero-inflated models have a zero part and a count part whereas nonzero-inflated models only have a count part. The dash line represents IRR=1 and error bars denote the 95% CI of the IRR. **(I)** Agreement analysis between ALICE and human counts using Gwet's AC1 for the 5 CTC phenotypes. Error bars represent the 95% CI.

Encouraged by ALICE's performance on synthetic images, we next sought to characterize the software CTC phenotyping enumeration using actual patient-derived fluorescent images and comparing the outcome against that of human enumeration. This comparison is necessary for the assessment of the potential application and reliability of ALICE in actual clinical settings. We selected two different sets of fluorescent images containing captured CTCs either with human epididymis secretory protein 4 (HE4+) or without HE4 (HE4-) from 61 ovarian cancer patients 42 and epithelial (*E*), hybrid (*H*) and mesenchymal (*M*) CTCs from 46 pancreatic cancer patients 43. ALICE was able to accurately identify and correctly locate these rare CTCs despite their extremely small numbers (< 0.1%) in the dense image set containing vast numbers of white blood cells (**Figure 4A-B**). Considering the rarity of CTCs (manifesting as a huge number of zeros in the CTC phenotypic count data), we opted to fit four different models: Poisson (P), negative binomial (NB), zero-inflated Poisson (ZIP) and zero-inflated negative binomial (ZINB) for ALICE versus human counts. The method of enumeration was entered into all 4 models as a factor with two levels: human enumeration and ALICE enumeration. Therefore, the manual count was taken as the reference and the ALICE count benchmarked against it.

Based on the lowest Akaike information criterion (AIC) values offered by the four models, the chosen regression models for HE4-, HE4+, *E*, *H* and *M* CTCs were respectively, ZIP, P, ZIP, ZIP and P (inset tables in **Figure 4C-G**). Further, from the incidence rate ratio (IRR) of the count part, the CTC counts by ALICE were found to be statistically indifferent from human counts for all 5 phenotypes, since all their IRR had 95% CI (*P* > 0.05) that crossed the value one (**Figure 4H** with details in **Table S7**). Likewise, the IRRs for the zero part of the regression models were statistically insignificant as well (**Figure 4H**), implying that the enumeration by ALICE will not increase the odds of observing an excess zero in the CTC counts. The agreement between ALICE and human counts based on Gwet's AC1 revealed that the Gwet's AC1 statistic for the HE4-, HE4+, *E*, *H* and *M* CTCs were 0.725 (95% CI: 0.652-0.789), 0.907 (95% CI: 0.819-0.955), 0.961 (95% CI: 0.918-0.983), 0.958 (95% CI: 0.925-0.978) and 0.884 (95% CI: 0.836-0.916), respectively (**Figure 4I**). The results indicated that the agreement between ALICE and human counts ranged from “substantial” to “almost perfect” in the Landis and Koch benchmark scale 44.

Next, it is most useful from a clinical point of view to provide a comparison of the time required by ALICE and human enumeration of CTC phenotypes in a real image set. For this purpose, we chose three CTC phenotypes: *E*-CTC, *H*-CTC and *M*-CTC sourced from 46 pancreatic ductal adenocarcinoma (PDAC) patients. For a human analyst, it generally takes 2-8 hours to count, typically, 80 FOVs per patient, with each FOV having 5 channels: bright field, nucleus marker DAPI, pan-leukocyte marker CD45, mesenchymal marker vimentin and epithelial marker E-cadherin. On the other hand, ALICE takes about an hour to complete an even more thorough scanning and enumeration task that included all the images of WBCs.

### A serendipitous encounter with circulating hybrid cells (CHCs) in PDAC patients

**Figure 5:**
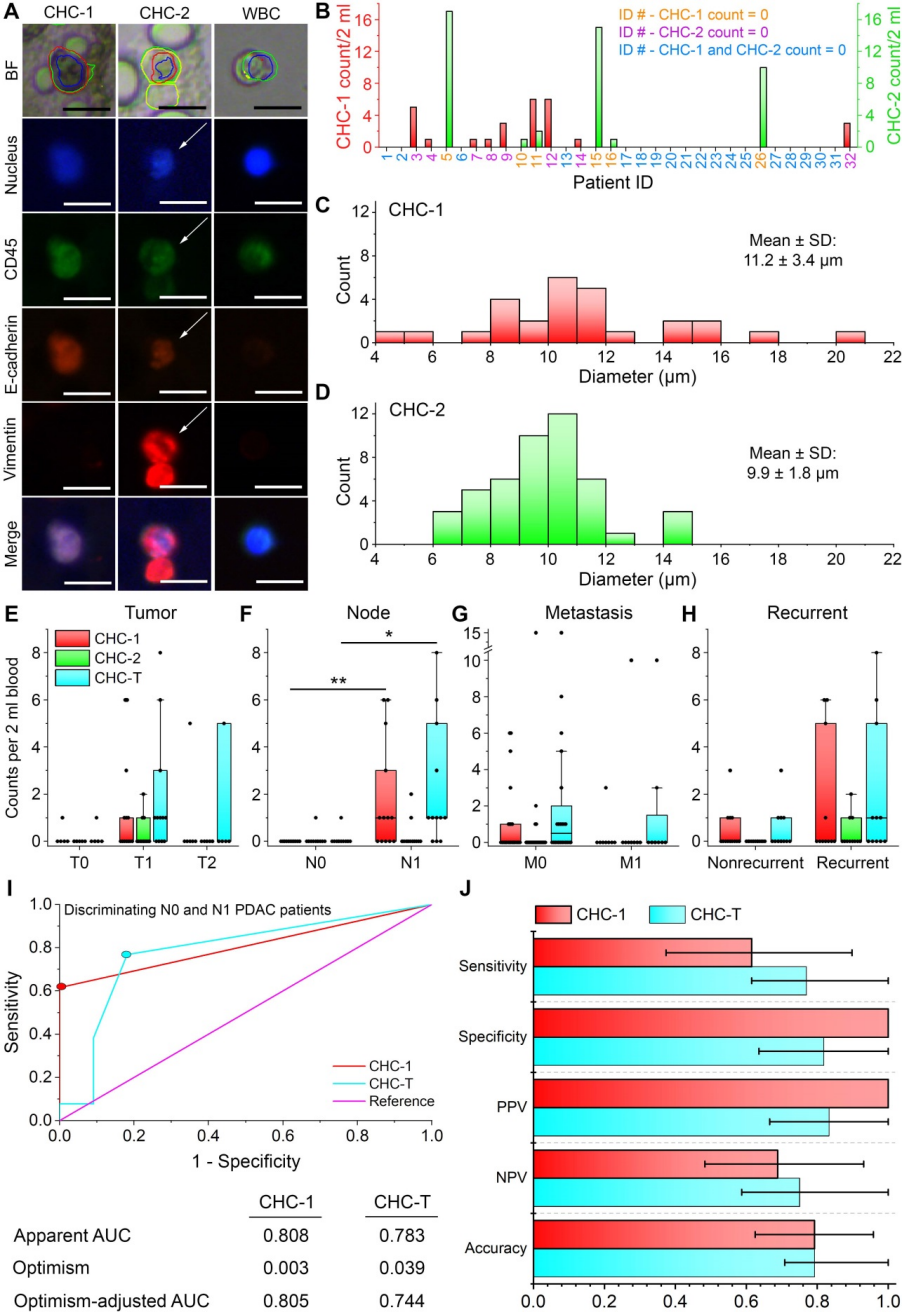
** Circulating hybrid cells (CHCs) in pancreatic cancer patients. (A)** Two populations of fusion hybrid identified by ALICE: CHC-1 (DAPI+/CD45+/E-cadherin+/vimentin-) and CHC-2 (DAPI+/CD45+/E-cadherin+/vimentin+) embedded in an overwhelming population of WBCs (DAPI+/CD45+/E-cadherin-/vimentin-) in pancreatic cancer patients. Scale bar: 20 µm. **(B)** Frequency histogram of CHC-1 and CHC-2 counts in pancreatic cancer patients. **(C-D)** Size distribution of CHC-1 and CHC-2. **(E-H)** Correlation of CHC-1, CHC-2 and CTC-T with T stage (*n*=24), N stage (*n*=24), M stage (*n*=32) and recurrence (*n*=32). * - *P* < 0.05; ** - *P* < 0.01 from Mann-Whitney U test. **(I)** Receiver operating characteristic (ROC) curves for CHC-1 and CHC-T in differentiating N0 and N1 PDAC patients with their respective apparent area under the curve (AUC), optimism and optimism-adjusted AUC calculated over 10000 bootstrap iterations. Colored dots represent the selected cutoff of 1 CHC-1/2 ml of blood and 1 CHC-T/2 ml of blood. **(J)** Validity of CHC-1 and CHC-T as PDAC node-positive biomarker in terms of the sensitivity, specificity, positive predicted value (PPV), negative predicted value (NPV) and accuracy. The error bars denote the 95% CI.

During ALICE's default full combinatorial scan of captured cell images in the peripheral blood of PDAC patients 43, we unexpectedly discovered a novel population of fusion hybrid cells that simultaneously express both hematopoietic (CD45) and tumor (E-cadherin) antigens. The classical enrichment technique for harvesting CTCs is to exclude the overwhelming presence of CD45+ expressing cells and this practice leads to an inadvertent preclusion of CHCs in human enumerative assays. We found 2 types of CHCs: CHC-1 (DAPI+/CD45+/E-cadherin+/vimentin-) and CHC-2 (DAPI+/CD45+/E-cadherin+/vimentin+) in our study (**Figure 5A**) and defined the CHC-Total (CHC-T) as the sum of the two fusion hybrids. The baseline characteristics of the 32 PDAC patients with 14 patients exhibiting positivity for CHC-T and 18 for the absence of CHC-T are listed in **Table 1**. Looking at the individual subpopulations of the rare fusion hybrid, we found that 8/32 (25.0%) patients had 1-6 CHC-1s/2ml of blood; 5/32 (15.6%) patients had 1-17 CHC-2s/2ml and 1 patient had both: 6 CHC-1s/2ml and 2 CHC-2s/2ml (**Figure 5B**). Further, the CHC-1 fusion hybrids possessed a more uniform size distribution with a min-max diameter range of 4 µm to 21 µm (mean (SD): 11.2 (3.4) µm) (**Figure 5C**). The CHC-2s on the other hand were narrower with a min-max diameter range of 6 µm to 15 µm (mean (SD): 9.9 (1.8) µm) (**Figure 5D**).

Interestingly, no correlation was observed between the CHC and CTC counts (**Table S8**). Also, there was no significant difference in the CHC-1, CHC-2 and CHC-T count when patients were stratified in accordance to their tumor size (**Figure 5E**). However, when stratified by the node stage, the CHC-1 and CHC-T counts were found to be significantly larger for patients with lymph node metastasis (N1) (*P* = 0.002 and *P* = 0.011, respectively) (**Figure 5F**). For the metastatic and recurrence status, once again, null results were obtained (**Figures 5G-H**). Based on these results, the receiver operating characteristic (ROC) curves for CHC-1 and CHC-T in discriminating N0 and N1 patients were drawn to evaluate the performance of these two counts as node-positive biomarkers (**Figure 5I**). To obtain a more accurate estimate of the two fusion hybrids' diagnostic performance, we have opted to report the optimism-adjusted area under the curve (AUC) 45. The optimism-adjusted AUCs of the CHC-1 and CHC-T were 0.805 and 0.744, respectively, and this indicated that the CHC-1 had a slightly better diagnostic ability in classifying N0 and N1 PDAC patients. By defining a cutoff of 1 CHC-1 per 2 ml of blood based on the highest Youden's index in **Figure 5I**, the leave-one-out cross-validated sensitivity = 0.615 (95% CI: 0.374-0.898), specificity = 1.000 (95% CI: 1.000-1.000), positive predictive value (PPV) = 1.000 (95% CI: 1.000-1.000), negative predictive value (NPV) = 0.688 (95% CI: 0.483-0.931) and accuracy = 0.792 (0.625-0.958). Likewise, a cutoff of 1 CHC-T per 2 ml of blood (again from the highest Youden's index) achieved a sensitivity = 0.769 (95% CI: 0.615-1.000), specificity = 0.818 (95% CI: 0.636-1.000), PPV = 0.833 (95% CI: 0.666-1.000), NPV = 0.750 (95% CI: 0.587-1.000) and accuracy = 0.792 (0.708-1.000) (**Figure 5J**; results of training data for CHC-1 and CHC-T are listed in **Table S9**).

## Discussion

ALICE is designed to automatically and simultaneously analyze and enumerate multiple cellular liquid biopsy phenotypes (up to 20 phenotypes) in fluorescent microscopy images, regardless of the image size. In addition to the count of a particular cell phenotype, ALICE also outputs the position and (optionally) thumbnails of the identified cells for further scrutiny. ALICE is configured for use across both the research and clinical settings. In the research setting, ALICE can be adopted by researchers as a standard pipeline for analyzing fluorescent images containing rare liquid biopsy cells such as CTCs and CHCs. ALICE not only increases the data analysis throughput but also, promotes an objective and repeatable research in the field of cellular liquid biopsies. In the clinical setting, the software can be used by hospitals for a rapid and reliable CTC and/or MTC analyses for disease management outcomes. Further, ALICE can be applied in the education sector for general pedagogy purposes (**Figure 1B**). A comparison of ALICE with other automated CTC detection software is listed in **Table 2.**

The novelty of ALICE lies not only with its state-of-the-art image processing capabilities but also, in reaching out to several user communities through an enhanced connectivity structure and in guarding against cyberthreats and adverse image manipulations via an advanced cybersecurity system. Taking hints from Google DeepMind gaming program, AlphaZero 46 that combined deep neural networks and traditional symbolic Monte Carlo tree search, we adopted a similar hybrid AI strategy in ALICE by integrating machine learning with rule-based algorithms to accurately enumerate various CTC phenotypes beyond the traditional confinement of counting only epithelial CTCs found in current software. In the first part of the hybrid AI, machine learning is employed to evaluate the threshold correction factor for ALICE to flexibly adapt and correctly process dense fluorescent images derived from disparate conditions of the cellular liquid biopsy. Trained and tested using a large number of images (>50 K) sourced from 6 synthetic and real image sets, the implemented regression model showed a low test error and a good generalization ability in handling images obtained under an assorted range of clinical environments that reflect the different microscopy systems of hospitals 47. In the second part of the hybrid AI, the rule-based algorithm enables ALICE to extend the identification, localization and enumeration process to use a full gamut of conventional and unconventional marker combinations to assay a diverse range of cell phenotypes (mesenchymal, hybrid, HE4 CTCs, CHCs, etc.).

ALICE is designed with an extended external connectivity to encourage a wide spectrum of appurtenant user communities to use the software. Linking electronic health records and the transfer of data and images across hospital communities can speed up the process of diagnosis, treatment and clinical management decision making 48. Likewise, the connectivity of ALICE can be exploited by the research community to foster effective and efficient collaborations. ALICE is also accessible by two other communities; education and public for the delivery of general and continuing education services and pedagogy. Different versions of specially configured ALICE are provided to connect with the four user communities. With the extended connectivity, ALICE needs to be safeguarded against intrusive hacking and malicious tampering of data and this scenario was vividly demonstrated by the use of fake CT scan images 49. ALICE is armed with a quad-layer protective feature for detecting and handling the majority of image forgeries encountered in digital image forensics 50. For example, the planting of fake or malicious images can be detected and removed to maintain the data integrity, as well as, the prevention of false results being generated from tampered images.

The fusion between immune and epithelial cells in tumors has already been widely reported 51-54 but this is not the case for mobile fusion cells in body fluids of cancer patients. The serendipitous discovery of CHCs in the peripheral blood of pancreatic cancer patients by ALICE raises the strong possibility of similar circulating hybrid cells in other epithelial cancers. Our CHCs are different from reported hybrid cells in blood 55, 56 in two aspects: our tumor cells expressed positivity to the pan-leukocyte antigen CD45 and either one of the combinations; CTC epithelial marker E-cadherin or simultaneously, both E-cadherin and the CTC mesenchymal marker vimentin. Several studies have suggested that the fusion of tumor and immune cells give rise to tumor heterogeneity that generates increased metastasis in pancreatic cancer patients 57. It was shown that cancer patients with CHCs have a poor prognosis 58-60 and correlates with the disease staging and overall survival 57. However, in our work, we found that the CHC-1 count was significantly higher for pancreatic cancer patients with N1 status compared to the N0 patients (**Figure 5F**). For the M stage, we did not find any significant difference in the CHC-1, CHC-2 and CHC-T counts (**Figure 5G**). This raises an interesting hypothesis whereby heterogeneity in the expression of the epithelial marker exists in CHCs that leads to different biological functions, much similar to that of conventional CTCs that express CK and EpCAM differently 55, 61, 62. The ROC and AUC analysis of CHC-1 and CHC-T indicated that these CHC populations can be a potential high specificity lymph node metastasis biomarker for pancreatic cancer (**Figure 5I-J**). Although promising for the diagnosis and treatment planning of PDAC patients 63-65, the results obtained in this study are still in the early stages and thus, a larger, prospective validation study of the node positive biomarker is warranted.

**Figure 6:**
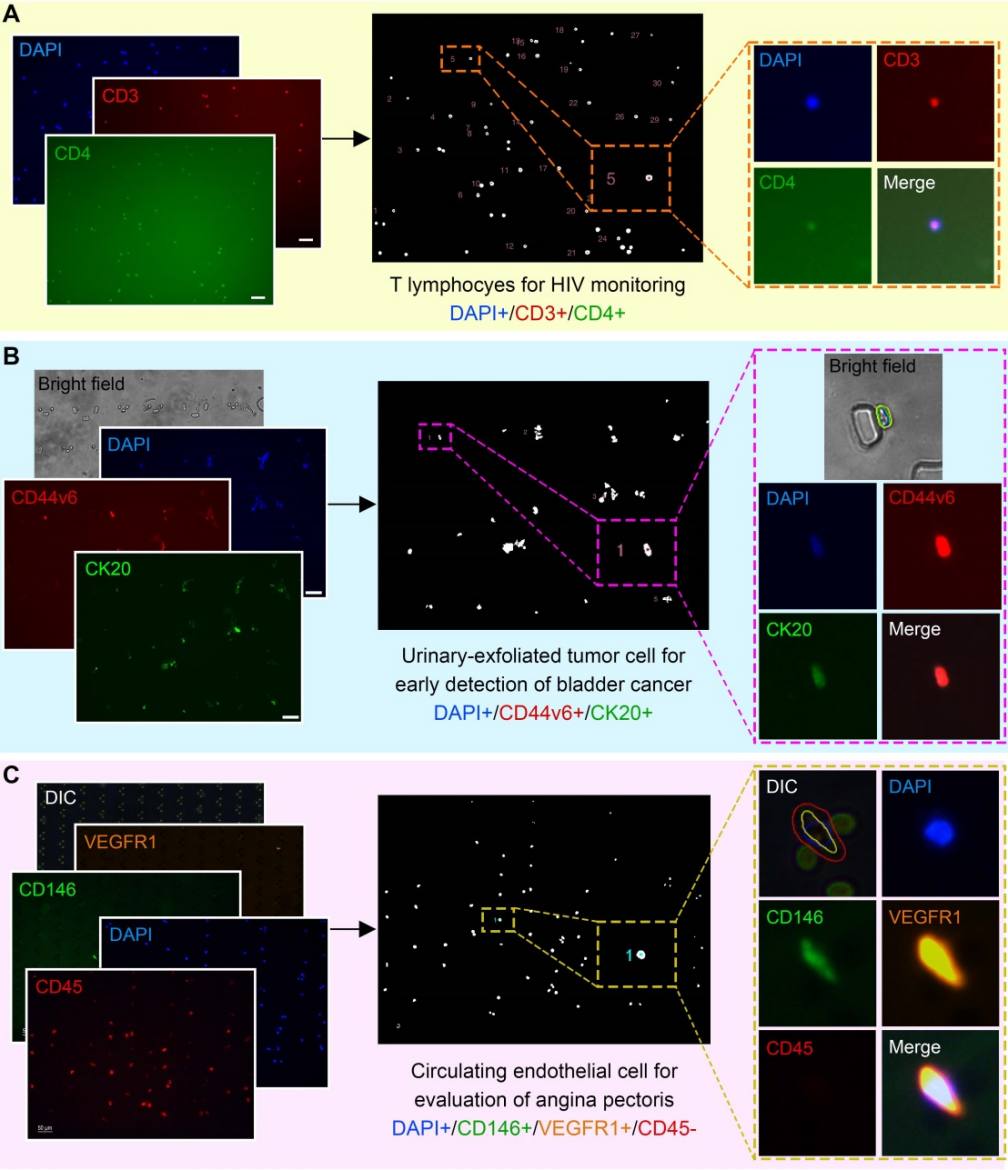
** ALICE for Non-CTC Cellular Liquid Biopsies: Identification, Localization and Enumeration. (A)** Enumeration of T lymphocytes for the monitoring of HIV patients. **(B)** Urinary-exfoliated tumor cells in bladder cancer patients for the early detection of bladder cancer. **(C)** Circulating endothelial cells in unstable angina and chronic stable angina patients for the evaluation of angina pectoris. For all three subpanels, the first column shows the raw input fluorescence images, the middle column depicts the nucleus mask image labeled with the identified cells and the last column highlights the exported cell thumbnail results of the identified cells by ALICE.

Although only validated and compared with CTC phenotypes in this study, ALICE has the potential to enumerate other cellular liquid biopsies such as the T lymphocytes 66 (**Figure 6A**), urinary-exfoliated tumor cell 67 (**Figure 6B**) and circulating endothelial cells 68 (**Figure 6C**) and this can accelerate the adoption of cellular liquid biopsies in other diseases. It is worth noting that the underlying algorithm implemented in ALICE can also be used for handling applications beyond traditional liquid biopsy to encompass the enumeration of general cellular phenotyping.

Being a cell-based liquid biopsy technology, ALICE may not be as sensitive as genomic-based liquid biopsy approaches that assays circulating tumor DNA (ctDNA) 69, 70. However, ALICE can first be used for a rapid identification of single-cell CTC phenotypes followed by a downstream genomic or transcriptomic analysis of captured CTCs in such a way that these two technologies can complement each other 71. For example, ctDNA analysis may be used to monitor cancer patients whereas single-cell CTC analysis can be used to reveal more actionable information to guide therapeutic decisions 72.

## Conclusions

We presented a hybrid AI algorithm developed using rule-based and machine-learning strategies for an automated and flexibly-adapting enumeration in cellular liquid biopsy applications. In particular, it is designed for a fast, accurate and reliable CTC phenotypic identification and quantitative assessment of several phenotypes. Implemented as ALICE, the software comes with enhanced cybersecurity and connectivity for use by medical researchers and members of the public alike. Further, our hybrid algorithm led to an unexpected discovery of CHC-1s (DAPI+/CD45+ /E-cadherin+/vimentin-) and CHC-2s (DAPI+ /CD45+/E-cadherin+/vimentin+). We showed that these CHCs can potentially be used as a high specificity biomarker for lymph node metastasis for pancreatic cancer.

## Methods

### Overall study design and sample selection

The objective of this retrospective study is to characterize and verify the performance of ALICE. Synthetic and real images were curated into different sets to handle contrasting usages that included training, validation and testing of regression models (12480 images), classification models for synthetic (12448 images) and tampered (46,936 images) input image detection, anomalous input image detection (400 images), final detection of a combination of synthetic, tampered and anomalous input images (1000 images), quantification of the accuracy and reliability of synthetic images (2000 images) and patient-derived real images (950 images), comparison with other bioimage software (744 images) and processing time characterization of ALICE (240 images). The images came from 14 different image sets with details as follows.

### Human HT29 colon cancer cells image set (BBBC001v1) 37

This image set consists of 6 FOVs of human HT29 colon cancer cells stained with Hoechst 33342 DNA stain. Each image contains 512 × 512 pixels. Counts from two different humans are provided and the mean of the two human counts is taken as the ground truth. This image set was used for the comparison of ALICE against other bioimage software.

### Drosophila Kc167 cells image set (BBBC002v1) 37

This image set has 5 different samples of *Drosophila melanogaster* Kc167 cells stained for DNA with Hoechst 33342. It contains a total of 50 images with 10 FOVs for each sample. The image size is 512 × 512 pixels. Similar to the previous image sets, the counts from two different humans are provided and the mean is taken as the ground truth. The image set was used for comparing ALICE against other bioimage recognition software.

### Synthetic cells image set (BBBC004v1) 73

This image set has 100 FOVs containing simulated objects with various degrees of overlapping and clustering using the SIMCEP simulating platform for fluorescent cell population images 74, 75. Each image is 950 × 950 in size. Data augmentation was performed by mirroring the image in the horizontal and vertical direction, as well as the combination of both directions to produce a total of 400 images (augmentation factor of 4). All 400 images were used for the training, validation and testing of the classification models for the detection of synthetic input images.

### Simulated high-content screening image set (BBBC005v1)

This image set contains simulated high-content screening (HCS) images using the SIMCEP simulating platform for fluorescent cell population with a clustering probability of 25% and a charge-coupled device (CCD) noise variance of 0.0001. Each image is 696 × 520 pixels and the whole image set has a total of 19,200 images (both in-focus and out-focus images). The nucleus counts for each of the FOVs are provided in 76. Four thousand and eight hundred in-focus FOVs (augmented from 1200 FOVs) were used for the training, validation and testing of the classification models for the detection of synthetic input images whereas only 2650 in-focus FOVs were used for to train, validate and test the regression models for the automatic determination of the threshold correction factor. A total of 560 FOVs were used to compare the performance between ALICE and other bioimage software. A further 60 FOVs were selected randomly for the characterization of the processing time of ALICE. Lastly, the provided binary masks were used to randomly segment up to 3 objects from the image in this set and pasted onto images from BBBC021v1, BBBC022v1 and BBBC038v1 sets to form the tampered images.

### Human U2OS cells image set (BBBC006v1)

The images were acquired from one 384-well microplate containing U2OS cells stained with the nucleus marker Hoechst 33342 and the cytoplasm marker phalloidin. Each image is 696 × 520 pixels and the whole image set contains 52,225 images generated from 32 z-step increments for each of the 768 FOVs. Similar to BBBC005v1, the ground truth for each FOV is provided in 76. A total of 2560 (augmented from 640 of the 768 in-focus FOVs) images were used for the training, validation and testing of the regression models implemented in ALICE. The rest of the FOVs (128 FOVs) were used to compare the performance between ALICE and other bioimage software.

### Human MCF7 cells compound-profiling experiment image set (BBBC021v1) 77

This image set contains 39600 FOVs of MCF7 breast cancer cells stained for DAPI, F-actin and B-tubulin (each channel having 13200 FOVs) and imaged by fluorescent microscopy after being treated with a collection of 113 small molecules at 8 different concentrations. Each image is 1280 × 1024 pixels. Two thousand one hundred and sixty images were randomly selected as the target images for the splicing operation in the creation of tampered images. A different set of 60 nuclei images were selected randomly for the characterization of ALICE's processing time.

### Human U2OS cell compound-profiling cell painting experiment image set (BBBC022v1) 78

This image set contains 69120 FOVs of U2OS cells treated with 1600 known bioactive compounds. With 5 channels for each FOV: con A, Hoechst 33342, MitoTracker Deep Red, WGA/phalloidin and SYTO 14 channels, this amounts to a total of 345600 fluorescent images in this image set. The images were captured using a 20X magnification and have a resolution of 696 × 520 pixels. A total of 2500 images were used as targets for the splicing operation in the creation of tampered images and another 916 images were used as the real/normal/pristine images in the final combined synthetic, anomalous and tampered detection image set.

### Human hepatocyte and murine fibroblast cells co-culture experiment image set (BBBC026v1) 79

This set contains images of co-cultured hepatocytes and fibroblasts in 384-well plate. There is a total of 864 FOVs of nuclei stained with Hoechst 33342 DNA stain and each image is 1392 × 1040 pixels. One thousand five hundred and sixty-eight (augmented from 392) images were used for the training, validation and testing of the classification models for the detection of synthetic input images. A further 60 nuclei images were selected randomly for the characterization of ALICE's processing time.

### Simulated 24-well plate with synthetic cells image set (BBBC031v1) 74

This synthetic HCS dataset was generated to simulate drugs perturbing the cell shape and expressions of proteins. The set contains 216 images with 9 images per well. The image size is 950 × 950 pixels. The provided binary masks were used to randomly segment up to 3 objects from the image in this set and pasted onto images from BBBC021v1, BBBC022v1 and BBBC038v1 image sets to form the tampered images.

### Kaggle 2018 Data Science Bowl Image Set (BBBC038v1)

This dataset consists of 670 FOVs of nuclei images created for the Kaggle 2018 Data Science Bowl. The nuclei were stained either fluorescently or histologically under various magnifications, quality of illumination, size of image, contexts including cell division, genotoxic stress, differentiation and others. The 545 fluorescently stained nuclei FOVs were extracted and among this, 1760 (augmented from 440) FOVs were used for the training, validation and testing of both the synthetic input image classification models and the threshold correction factor regression models. This image set also serves as the target images for the splicing operation in the creation of tampered images.

### Nuclei of U2OS cells in a chemical screen image set (BBBC039v1) 80

This image set contains 200 FOVs of nuclei stained with the Hoechst stain and imaged using a fluorescence microscope during a high-throughput chemical screen on U2OS cells. The nuclei images in this image set present a variety of nuclear phenotypes. All the FOVs (total of 800 FOVs; augmentation of 4) were used for the training, validation and testing of the machine learning regression models implemented in ALICE. Another 100 images were chosen as normal images in the testing of the anomaly detection algorithm.

### Synthetic fluorescent cell phenotypes image set

The synthetic fluorescent cell image set was created using SimuCell. Four groups of synthetic fluorescent images were simulated with one group containing 100 FOVs of 5 randomly selected phenotypes. Within each FOV, there is one nucleus image (Type: “nucleus”, Model: “Nucleus_Model”, nuclear radius: 13, nuclear eccentricity: 0.5, extent of variation: 0.1) and either without any cytoplasm images or having up to 3 cytoplasm images (Type: “cytoplasm”, Model: “Centered_Cytoplasm_Model”, cell radius: 18, cell eccentricity: 0.5, extent of variation: 0.3, centered around: Nucleus). A constant marker level and standard deviation of 0.5 and 0.2, respectively, were used for all images, along with the addition of Perlin texture for a realistic representation of the marker expression. The number of the five phenotypes in each FOV was randomly generated. In total, this image set contains 400 FOVs and 2000 individual synthetic fluorescent images. This image set was used for the performance assessment of ALICE. A further randomly selected 1280 images were used for the training, validation and testing of the classification models for the detection of synthetic input images.

### Captured cells of ovarian cancer patients' image set 42

This image set contains fluorescent images of captured cells from the blood of ovarian cancer patients recruited from Peking University People's Hospital with protocols approved by the hospital's institutional review boards and written informed consent was obtained from all patients. CTCs were captured using a specially designed microfluidic chip - *triangular unit* (TU)-chip^TM^ with 8 capture chambers × 693 capture unit (CU)/chamber = 5544 CUs, each consisting of a group of 3 elliptical micropillars placed in a triangular configuration (**Figure S5**). Two ml of blood was obtained from each patient, centrifuged to discard the serum and diluted with a buffer solution of volume ratio 1:1. Next, the diluted blood was syringe-pumped into the microfluidic chip at a flow rate of 500µl/h. Captured cells were fixed with 4% paraformaldehyde for 15 min, permeabilized with 0.1% Triton X-100 for 10 min, washed with phosphate buffered saline (PBS) for 20 min and perfused with 5% BSA for 30 minutes to prevent nonspecific binding of antibodies. The cells were then stained with DAPI (Molecular probes, D1306), Alexa Fluor 488 conjugated anti-CD45 (Invitrogen, MHCD4520), phycoerythrin conjugated anti-EpCAM (Abcam, ab112068), phycoerythrin conjugated anti-panCK (Abcam, ab52460), phycoerythrin conjugated anti-vimentin (Abcam, ab209446), phycoerythrin conjugated CK7/17 (Novus Biologicals, NB500-352PE) and unconjugated anti-HE4 (Abcam, ab200828) with secondary donkey anti-rabbit Alexa Fluor 647 (Abcam, ab150067). There is a total of 130 FOVs and each FOV has 5 channels: one bright field, one nucleus channel (DAPI), one epithelial and mesenchymal CTC marker channel (E&M; EpCAM, panCK, vimentin and CK7/17), one leukocyte marker channel (CD45) and one ovarian specific marker channel (HE4). Hence, the total number of images amounted to 650. Each image is 2560 × 1920 pixels. Two CTC phenotypes were defined based on the expression of HE4, either HE4- CTC with DAPI+/E&M+/CD45-/HE4- or HE4+ CTC with DAPI+/E&M+/CD45-/HE4+. The counts of trained human analysts were taken as the ground truth. This image set was used for a comparison of ALICE and human count of CTC phenotypes. A further 1424 randomly selected fluorescent images were used for training, validation and testing of the classification models for the detection of synthetic input images.

### Captured cells of pancreatic cancer patients' image set 43

This image set contains fluorescent images of captured cells from the blood of pancreatic adenocarcinoma (PDAC) patients recruited from Peking Union Medical College Hospital with approved protocols by the institutional review boards and written informed consent was obtained from all patients. Similar to the ovarian cancer samples, 2 ml of blood was drawn and processed using the TU-chip^TM^ as well with the only exception of using a faster flow rate of 2 ml/h. Next, the captured cells were first washed with PBS, fixed with a 1% paraformaldehyde flow for 15 min, washed with PBS for 10 min, permeabilized with 0.1% Trixon X-100 for 15 min, washed again with PBS for 10 min and blocked with BlockAid Blocking Solution (Life Technologies, B1070) for 30 min. Lastly, the captured cells were stained with DAPI (Molecular probes, D1306), Alexa Fluor 488 conjugated anti-CD45 (Invitrogen, MHCD4520), Alex Fluor 555 conjugated anti-E-cadherin (Abcam, ab206878), Alexa Fluor 647 conjugated anti-vimentin (Abcam, ab195878), Alexa Fluor 488 conjugated anti-E-cadherin (Abcam, ab185013), Alexa Fluor 555 conjugated anti-vimentin (Abcam, ab203428) and Alexa Fluor 647 conjugated anti-CD45 (Abcam, ab200317). There is a total of 377 FOVs and each FOV has 5 channels (total of 1885 images): one bright field, one nucleus channel (DAPI), one epithelial CTC marker channel (E-cadherin), one mesenchymal CTC marker channel (vimentin) and one leukocyte marker channel (CD45). Each image is 2560 × 1920 pixels. Based on this, three CTC phenotypes were defined: epithelial CTC (*E*-CTC) with DAPI+/CD45-/E-cadherin+/vimentin-, mesenchymal CTC (*M*-CTC) with DAPI+/CD45-/E-cadherin-/vimentin+ and hybrid CTC (*H*-CTC) with DAPI+/CD45-/E-cadherin+/vimentin+. The counts of trained human analysts were taken as the ground truth. Five thousand one hundred and twenty (augmented from 1280) and 1216 (augmented from 304) images from this set were used to train, validate and test the regression models and synthetic image classification models, respectively. The rest of the images were used for the comparison of ALICE performance against human analysts. A further 60 FOVs were selected randomly for characterizing the processing time of ALICE.

### Feature extraction

Two different groups of features were extracted from each preprocessed image. The first group pertained to the statistical information of the whole image histogram which included the mean, standard deviation, skewness, kurtosis, energy, entropy and smoothness, as previously defined 81. Besides that, the image was split into 3 × 3 blocks of subimages and the same seven statistics were computed for each subimages in order to extract the spatial information of the images. Thus, the total number of features extracted for the first group was 7+7×9 =70. The 2^nd^ group involved the Gabor features, which are features extracted after applying Gabor filters to the image. A total of 24 Gabor filters were employed using a combination of four different scales (2.0, 2.5, 3.0 and 3.5 pixel/cycle) and 6 different directions (0°, 60°, 120°, 180°, 240° and 300°). Next, the mean, variance, skewness and kurtosis of the Gabor-transformed image were extracted as the Gabor features, resulting in a total of 24×4 = 96 Gabor features per image. In the end, the Gabor features and the histogram statistical features were combined into one feature vector containing 166 features for each image.

### Machine learning models in ALICE

Both regression and classification machine learning models are available in ALICE, with the former models used for the automated selection of threshold correction factors whereas the latter used for the automated detection of tampered and synthetic images. The available regression models in ALICE include linear (linear and robust terms), SVM (linear, quadratic, cubic, fine Gaussian, medium Gaussian and coarse Gaussian kernel functions), ensemble (boosting and random forest) and Gaussian process regression (GPR) (squared exponential, Matern 5/2, exponential, rational quadratic kernel functions). On the other hand, the classification models include decision tree (fine, medium and coarse trees), linear discriminant analysis, logistic regression, SVM (linear, quadratic, cubic, fine Gaussian, medium Gaussian and coarse Gaussian kernel functions), K-nearest neighbours (KNN) (fine, medium, coarse, cosine, cubic and weighted kernel functions) and ensemble (boosted, random forest, subspace discriminant, subspace KNN and RUSBoosted kernel functions). All models were trained and implemented in Matlab with default hyperparameter settings. The train and test sets were randomly split via a 80:20 ratio. The performance metrics for the machine learning models (using RMSE and MAE for regression models; sensitivity, specificity and accuracy for classification models) were evaluated on both sets by calculating the 10 × 10 fold cross-validation results on the training set and the test results on the test set.

### Thresholding methods tested for ALICE

For the development of ALICE, 16 different thresholding methods were tested and the best thresholding method was chosen and implemented in ALICE.

### Huang's fuzzy thresholding (Huang) 82

This thresholding method utilizes the measures of fuzziness of an image to identify an appropriate thresholding value that minimizes the fuzziness of the image.

### Alternative implementation of Huang's fuzzy thresholding (Huang2)

This is an alternative implementation of Huang's method with faster processing time when applied on 16-bit images.

### Intermodes thresholding (Intermodes) 83

Two local maxima will first be found by iteratively smoothing the image histogram, then the thresholding value was computed by taking the average of the two maxima pixel values.

### Iterative selection thresholding (Iterative Selection) 84

This method first involves creating a binary image using an initial thresholding value and then the average of the background and foreground pixels are calculated to produce a new thresholding value. This process is repeated iteratively until the binary image produced remains the same for further iterations.

### Li's minimum cross entropy thresholding (Li) 85

This method computes the thresholding value by minimizing the cross entropy between the original image and the thresholded image using the one-point iteration method.

### Maximum entropy thresholding (Maximum Entropy) 86

The thresholding value as determined by the maximum entropy algorithm is based on the maximization of the entropy between the foreground and background pixels.

### Mean of grey levels thresholding (Mean) 87

This simple algorithm determines the thresholding value by taking the average of grey levels.

### Minimum error thresholding (Minimum Error) 88

This algorithm aims to minimize the average pixel classification error rate under the assumption of a normally distributed histogram to find the thresholding value.

### Minimum thresholding (Minimum) 83

Similar to the Intermodes thresholding, two local maxima will first be found using the same method and the thresholding value is determined as the minimum point between the maxima.

### Moment-preserving thresholding (Moments) 89

The thresholding value for this method is computed deterministically such that the moments of an input image histogram is preserved in the output image.

### Otsu's threhsolding (Otsu) 90

The Otsu's method first searches exhaustively for all possible thresholding values and the value that minimizes the intra-class variance, defined as the weighted sum variances of the foreground and background, will be selected.

### Percentile thresholding (Percentile) 91

50% of the pixels are assumed to be the foreground pixels and the thresholding value will be chosen accordingly.

### Maximum Renyi entropy thresholding (Renyi Entropy) 86

This method is the same as the maximum entropy method with the exception that the Renyi entropy is used instead of the Shannon entropy.

### Shanbhag's thresholding (Shanbhag) 92

The original image is viewed to compose of two fuzzy sets, i.e. each pixel can have fractional membership values. The thresholding value will be determined by minimizing the information measure between the foreground and background.

### Triangle thresholding (Triangle) 27

A line connecting between the maximum and minimum of the input image histogram will first be constructed and the perpendicular distance from this line to all the values between the maximum and minimum will be calculated. The point with the maximum distance is chosen as the thresholding value.

### Yen's thresholding (Yen) 93

The thresholding value is determined by minimizing the maximum correlation criterion that factors in both the discrepancy between the thresholded and original image as well as the number of bits required to represent the thresholded image.

### Filtering of non-nucleic objects and creation of cytoplasmic masks

For the binarized nucleus image, non-nucleic objects will be filtered from the nucleus binary image based on the morphological characteristics of size and eccentricity (eccentricity of 0 represents a circle whereas an eccentricity of infinity represents a line). The rationale behind choosing these characteristics is because a cell nucleus has a finite range of sizes and has an oval shape in general. Any object in the nucleus binary image that has an area of < 200 pixels, > 2000 pixels or an eccentricity > 0.8 will be removed. For binarized cytoplasm marker images, the required cytoplasmic masks are created by multiplying together the appropriate combinations of binary cytoplasm images. If the non-expression of a particular marker is needed, then that particular image will first be inverted before creating the cytoplasmic mask.

### Enumeration of cell phenotypes

Before enumeration, a two-step filtering procedure was applied. The first step involved in the filtering uses the same criteria as the binary nucleus image to remove any debris pixels. The second filtering step used the growing algorithm of a region to detect and remove any objects that have a nucleus size larger than the cytoplasm. The initial step of the region growing algorithm was to locate the centroid of a particular object in the *masked* nucleus image and this point will serve as the seed for region growing until the border of the object was detected. Next, the area of the detected object in the *masked* nucleus image was calculated. The same two steps were again performed for the *filtered* nucleus binary image using the same point as the seed and the area was calculated as well. If the ratio of the two objects' area (masked/filtered) is smaller than 1, then this represents that the nucleus is larger than the cytoplasm. To be conservative, only objects with an area ratio of < 0.6 was removed. This comparison of the area ratios will be performed for all objects in the masked image. After completing the two-step filtering process, the enumeration of the remaining cells was done using a blob detection algorithm. The location and the number of blobs will be labeled with unique colors on the filtered nucleus binary image.

### Anomaly detection among input images

Anomalies in the input images are detected using robust principal component analysis (PCA) 30. In brief, based on the extracted feature list of the input images, robust PCA combines projection pursuit techniques and robust covariance estimation in order to group the input images into four possible categories: regular observation, good leverage points, orthogonal outliers and bad leverage points as determined by the points' orthogonal distance and score distance (see 30 for more details). In ALICE, input images that are categorized as bad leverage points are defined as anomalies and will be flagged. This (optional) step is performed separately for each of the channels.

### Localization of tampered region

The localization of possible tampered regions will only be done if two conditions are met: 1) the input image is deemed as tampered by ALICE and 2) enabled by the user. First, as proposed by Zeng, Zhan, Kang and Lin, the input image was segmented into 64 × 64 blocks as well as 32 × 32 blocks using a coarse-to-fine strategy and the noise level of each block was estimated using PCA 33. Instead of the proposed use of K-means clustering algorithm to group the blocks into either pristine or tampered regions (binary attribution), we implemented and modified the algorithm of Hosseini and Kirchner 32 that allows non-binary attribution clustering by using hierarchical clustering. More specifically, the optimal/correct number of clusters was assumed to range from 1 to 5 and was automatically chosen based on the largest gap statistic and largest silhouette index, respectively, for the clustering of the 64 × 64 blocks and the 32 × 32 blocks. The rationale of choosing the gap statistic for the former was to allow for the possibility of clustering into only 1 class (i.e. all pristine or all tampered) whereas the silhouette index for the latter case was chosen because this particular clustering validity index is considered to be one of the best 94. The class with the least number of pixels was considered to be tampered.

### Exportation of results

The labeled image and an Excel file containing the counts of each chosen cell phenotypes for each FOV will be exported to the output location specified by the user. Besides that, the user can also opt to export the thumbnails of the detected cell phenotypes and a bright field image containing the outline of each marker (if applicable). The exported thumbnails have a resolution of about 120 × 120 pixels. The exporting of these results allows the user to perform post-correction to the result or for other downstream analyses. Further, results pertaining to the flagging of anomalous, synthetic and tampered images can be optionally exported as well.

### Characterization of ALICE processing time

For each image size, the time needed to process and enumerate 20 FOVs were analyzed for the P1 phenotype and the experiment was performed in triplicate. The experiment was performed using a 64-bit desktop computer with Intel Core i7-8700 CPU @ 3.20 GHz, 3.19 GHz and 32 GB RAM.

### Simulation of realistic synthetic fluorescent images

In order to create synthetic images that are similar to the images in the BBBC022 image set, the average number of cells in 50 images in the image set and the distribution of the radius and eccentricity of 500 cells were evaluated (**Figure S6A-B**). Based on this information, 3 nucleus populations with different nucleus radius were defined: 15.7, 20.3 and 11.1 whereas the nuclear eccentricity and the extent of variation were set to 0.77 and 0.10, respectively, for all three populations in Simucell 34. In terms of the marker settings, all 3 populations had the same settings: mean marker level = 0.65, marker level standard deviation = 0.15, multiplicative Perlin texture, noise amplitude = 0.2, length scale = 6, falloff frequency = 0.0025 and noise type of standard 1/f. Twelve of such images were simulated. Examples of the simulated images are shown in **Figure S6C-E**.

### Statistical analysis

Statistical analysis was performed using SPSS version 24 (SPSS, Inc., Chicago, IL, USA) and MATLAB version 2018b (The MathWorks, Inc., MA, USA). Variables were first checked for normality using the Shapiro-Wilk test. Inferential analyses on the various machine learning models were performed by calculating the mean of the performance metrics (RMSE and MAE for the regression models; sensitivity, specificity and accuracy for the classification models) and then, ranking these models in accordance with the mean value 95, 96.

For benchmarking purposes, the methodology proposed by the Broad Institute was adopted such that the percentage mean error between ALICE's count and the ground truth was calculated. Further, the correlation between the ground truth and ALICE's count was analyzed using Passing-Bablok regression. The assumptions of correlation and linear relationship were tested by Kendall's tau coefficient and the cumulative sum linearity test, respectively, prior to the Passing-Bablok regression. A visual analysis of the agreement was performed using the Bland-Altman plot by graphing the difference between the two counts against the mean of the two counts.

A nonlinear curve fitting procedure was first applied to the processing time of ALICE with respect to the different image sizes in order to reveal the relationship between the processing time and the image size. Next, the mean processing time with/without parallel processing and exporting the results under fixed image size were compared using repeated measures analysis of variance (ANOVA). If a significant result is obtained, a planned comparison with Bonferroni correction (corrected *P* = 0.025) was performed for the post-hoc analysis such that comparisons were only done for the following 4 pairs: i) “No parallel, no export” vs “No parallel, export”, ii) “No parallel, no export” vs “Parallel, no export”, iii) “No parallel, export” vs “Parallel, export” and iv) “Parallel, no export” vs “Parallel, export”. The comparison of “No parallel, no export” vs “Parallel, export” and “No parallel, export” vs “Parallel, no export” pairs were not done as they are a comparison between 2 factors simultaneously.

For the comparison between human and ALICE's counts on CTC phenotypes, owing to a large number of zeros in the count data, four different regression models, namely the Poisson regression model, negative binomial (NB) regression model, zero-inflated Poisson (ZIP) regression model and the zero-inflated negative binomial (ZINB) regression model were fitted to the data. The method of enumeration was entered into all of the 4 models as a factor with two levels: human manual enumeration and software enumeration. The model with the lowest AIC was selected for further interpretation. An agreement analysis between ALICE and human counts was performed using the Gwet's AC1 method.

The counts of CHC-1, CHC-2 and CHC-T were compared between groups either using the Kruskal-Wallis test or the Mann-Whitney U test. Receiver operating curves (ROCs) were drawn and the corresponding optimism-adjusted AUC was calculated via the bootstrap validation technique 45 in order to assess the internal validity of the biomarkers. Briefly, a bootstrap sample was created and a binary logistic regression model was fitted to the bootstrap sample, followed by the calculation of the AUC. The same model was then applied to the original sample and a second AUC was calculated. The difference between these two AUCs is defined as the optimism. The average optimism over 10000 iterations was obtained and subtracted from the unadjusted AUC to get the optimism-adjusted AUC. This optimism-adjusted AUC represents a more accurate estimation of the diagnostic model's performance in new, unseen datasets. The optimal cutoff point for each of the three CHCs was chosen based on the highest Youden's index and the corresponding sensitivity, specificity, PPV, NPV and accuracy were calculated and validated using the leave-one-out cross validation (LOOCV) technique. The 95% confidence interval (95% CI) was computed using 1000 bootstrap iterations. A two-sided *P* < 0.05 was considered to be statistically significant.

## Supplementary Material

Supplementary figures and tables.

## Figures and Tables

**Table 1:** Baseline Patient Characteristics Stratified by CHC-T Positivity

Patient characteristic	CHC-T negative (n=18)	CHC-T positive (n=14)	*P* value
Age, mean (SD)	59.3 (7.8)	58.6 (9.0)	0.814
Males, No. (%)	6 (33)	9 (64)	0.082
Albumin, mean (SD), g	43.1 (3.3)	41.6 (3.6)	0.234
CA19-9 serum, median (IQR), U/ml	122 (28-174)	104 (70.8-412)	0.531
CEA serum, median (IQR), U/ml	2.4 (2.0-5.1)	3.6 (2.2-8.4)	0.368
CA242 serum. Median (IQR), U/ml	23.7 (5.7-70.5)	39.2 (19.5-142.8)	0.263
Location of tumor: head/body or tail, No. (%)	11 (61) / 7 (39)	11 (79) / 3 (21)	0.446
Tumor size, median (IQR), No. (%)	3.3 (2.0-4.8)	3.0 (2.5-3.9)	0.706
**T stage, No. (%)**			0.112
T0	4 (22)	1 (7)	
T1	4 (22)	9 (64)	
T2	4 (22)	2 (14)	
Tx	6 (34)	2 (14)	
**N stage, No. (%)**			0.007
N0	9 (50)	2 (14)	
N1	3 (17)	10 (72)	
Nx	6 (33)	2 (14)	
**M stage, No. (%)**			0.412
M0	12 (67)	12 (86)	
M1	6 (33)	2 (14)	
**TMN stage, No. (%)**			0.275
I	1 (6)	0 (0)	
II	11 (61)	12 (86)	
IV	6 (33)	2 (14)	
**Differentiation grade, No. (%)**			0.448
Well	1 (6)	0 (0)	
Moderate	7 (39)	7 (50)	
Poor	4 (22)	5 (36)	
Not specified	6 (33)	2 (14)	
**Perineural invasion, No. (%)**			0.421
No	3 (17)	4 (29)	
Yes	9 (50)	8 (57)	
Not specified	6 (33)	2 (14)	
**Perivascular invasion, No. (%)**			0.154
No	10 (56)	12 (86)	
Yes	2 (11)	0 (0)	
Not specified	6 (33)	2 (14)	
**Carcinoma cell embolus, No. (%)**			0.297
No	10 (56)	8 (57)	
Yes	2 (11)	4 (29)	
Not specified	6 (33)	2 (14)	
**Surgery, No. (%)**			0.412
Whipple	8 (44)	9 (64)	
Distal pancreatectomy	4 (22)	2 (14)	
Palliative surgery	2 (11)	1 (7)	
Others	1 (6)	2 (14)	
No surgery	3 (17)	0 (0)	
**Resection margin, No. (%)**			0.333
R0	5 (28)	7 (50)	
R1/R2	7 (39)	5 (36)	
Not specified	6 (33)	2 (14)	
**Chemotherapy, No. (%)**			0.425
No	4 (22)	3 (21)	
Yes	12 (67)	11 (79)	
Not specified	2 (11)	0 (0)	
**Number of CTCs/2 ml**			
Epithelial CTCs, mean (SD)	8.3 (8.0)	8.2 (6.7)	0.965
Mesenchymal CTCs, mean (SD)	19.8 (13.5)	17.2 (11.4)	0.573
Hybrid CTCs, mean (SD)	14.1 (12.2)	10.6 (7.5)	0.349
Total CTCs, mean (SD)	42.2 (30.7)	36.0 (24.1)	0.538

**Table 2:** Comparison of ALICE to other automated CTC detection software

Software	Algorithm	Advantage	Disadvantage
**PACE** (Precise and Automatic CTC Enumeration) 14	Morphological operations based on cell area, length/width ratio and circularity at specific regions of the microfluidic chip	• Detection of fluorescently stained CTCs; • Computationally efficient by processing only specific regions of the microfluidic chip.	• Unable to detect H&E stained CTCs; • Only able to detect epithelial CTCs; • Used in conjunction with specific microfluidic chip.
**ACCEPT** (Automated CTC Classification, Enumeration and Phenotyping) 22, 23	Deep learning algorithm via an epithelial marker staining	• Detection of fluorescently stained CTCs; • Open-source.	• Unable to detect H&E stained CTCs; • Only able to detect epithelial CTCs.
**CTC AutoDetect 1.0 24**	Morphological operations based on cell diameter and shape at 10 different focal lengths	Detection of H&E stained CTCs	• Unable to detect fluorescently stained CTCs; • Unable to differentiate CTC phenotypes.
**ALICE** (Automated Liquid Biopsy Cell Enumerator)	Hybrid AI that combines rule-based morphological operations and statistical machine learning algorithm	• Detection of fluorescently stained CTCs; • Able to enumerate up to 20 cell phenotypes simultaneously; • Built-in cybersecurity and connectivity functions.	• Unable to detect H&E stained CTCs; • Computationally expensive.

## Abbreviations

ACCEPT: Automated CTC Classification,Enumeration & PhenoTyping
AI: artificial intelligence
AIC: Akaike information criterion
ALICE: Automated Liquid Biopsy Cell Enumerator
ANOVA: analysis of variance
AUC: area under the curve
CAD: computer-aided diagnosis
CCD: charged-couple device
CHC: circulating hybrid cell
CK: cytokeratin
CLAHE: contrast-limited adaptive histogram equalization
CTC: circulating tumor cell
ctDNA: circulating tumor DNA
DAPI: 4',6-diamidino-2-phenylindole
E-CTC: epithelial CTC
EpCAM: epithelial cell adhesion molecule
FOV: field of view
GPR: Gaussian process regression
GUI: graphic user interface
H-CTC: hybrid CTC
H&E: hematoxylin & eosin
HCS: high-content screening
HE4: human epididymis protein 4
IOT: internet of things
IRR: incidence rate ratio
KNN: K-nearest neighbours
LOOCV: leave-one-out cross-validation
M-CTC: mesenchymal CTC
MCC: Matthews correlation coefficient
MTC: mobile tumor cell
NB: negative binomial
NPV: negative predictive value
P: Poisson
PACE: Precise and Automatic CTC Enumeration
PBS: Phosphate buffered solution
PCA: principal component analysis
PDAC: pancreatic ductal adenocarcinoma
PPV: positive predictive value
PSA: prostate-specific antigen
R, S, N, P, A, T: Real, synthetic, normal, Pristine, anomalous, tampered (images)
ROC: receiver operating characteristic
SVM: support vector machine
WBC: white blood cell
ZINB: zero-inflated negative binomial
ZIP: zero-inflated Poisson
