# Supplementary material for: ALICE: a hybrid AI paradigm with enhanced connectivity and cybersecurity for a serendipitous encounter with circulating hybrid cells
Source: Theranostics. 2020 Sep 2;10(24):11026–48. doi: 10.7150/thno.44053 (PMC7532685; doi:10.7150/thno.44053)
Supplement: Supplementary file 1 — Supplementary figures and tables. [file thnov10p11026s1.pdf]

SUPPLEMENTARY DATA

ALICE: a hybrid AI paradigm with enhanced connectivity and cybersecurity for a serendipitous encounter with circulating hybrid cells

Kok Suen Cheng, Rongbin Pan, Huaping Pan, Binglin Li, Stephene Shadrack Meena, Huan Xing, Ying Jing Ng, Kaili Qin, Xuan Liao, Zhipeng Wang and Ray P.S. Han

Supplementary Figures

No	Title
Figure S1	Testing of various thresholding methods.
Figure S2	Graphic user interface of ALICE.
Figure S3	Agreement analysis via Passing-Bablok regression for the remaining 16 phenotypes.
Figure S4	Agreement analysis via Bland-Altman plot for the remaining 16 phenotypes.
Figure S5	Design of the TU-chip™ and the experimental setup.
Figure S6	Creation of a realistic synthetic fluorescent image.

Supplementary Tables

No	Title
Table S1	Optimizing machine learning models for an automated selection of threshold correction factors.
Table S2	Definition of the 20 Phenotypes in ALICE (Automated Liquid Biopsy Cell Enumerator).
Table S3	Performance of the input image anomaly detection using robust principal component analysis (PCA) with varying combinations of parameters k and α under 4 different percentage of anomalies.
Table S4	Optimizing machine learning models for an automated detection of tampered input images.
Table S5	Optimizing machine learning models for an automated detection of synthetic input images.
Table S6	Comparison of classifiers in detecting synthetic images in the final testing dataset.
Table S7	Regression analysis of the counts of five different circulating tumor cell (CTC) phenotypes obtained from ALICE (Automated Liquid Biopsy Cell Enumerator).
Table S8	Correlation matrix of circulating hybrid cells (CHCs) and circulating tumor cells (CTCs) (Spearman's ρ and P value in parenthesis).
Table S9	Diagnostic performance of circulating hybrid cell (CHC)-1 and CHC-Total (CHC-T) in differentiating pancreatic ductal adenocarcinoma (PDAC) patients with lymph node metastasis in the training dataset.

Supplementary Figures

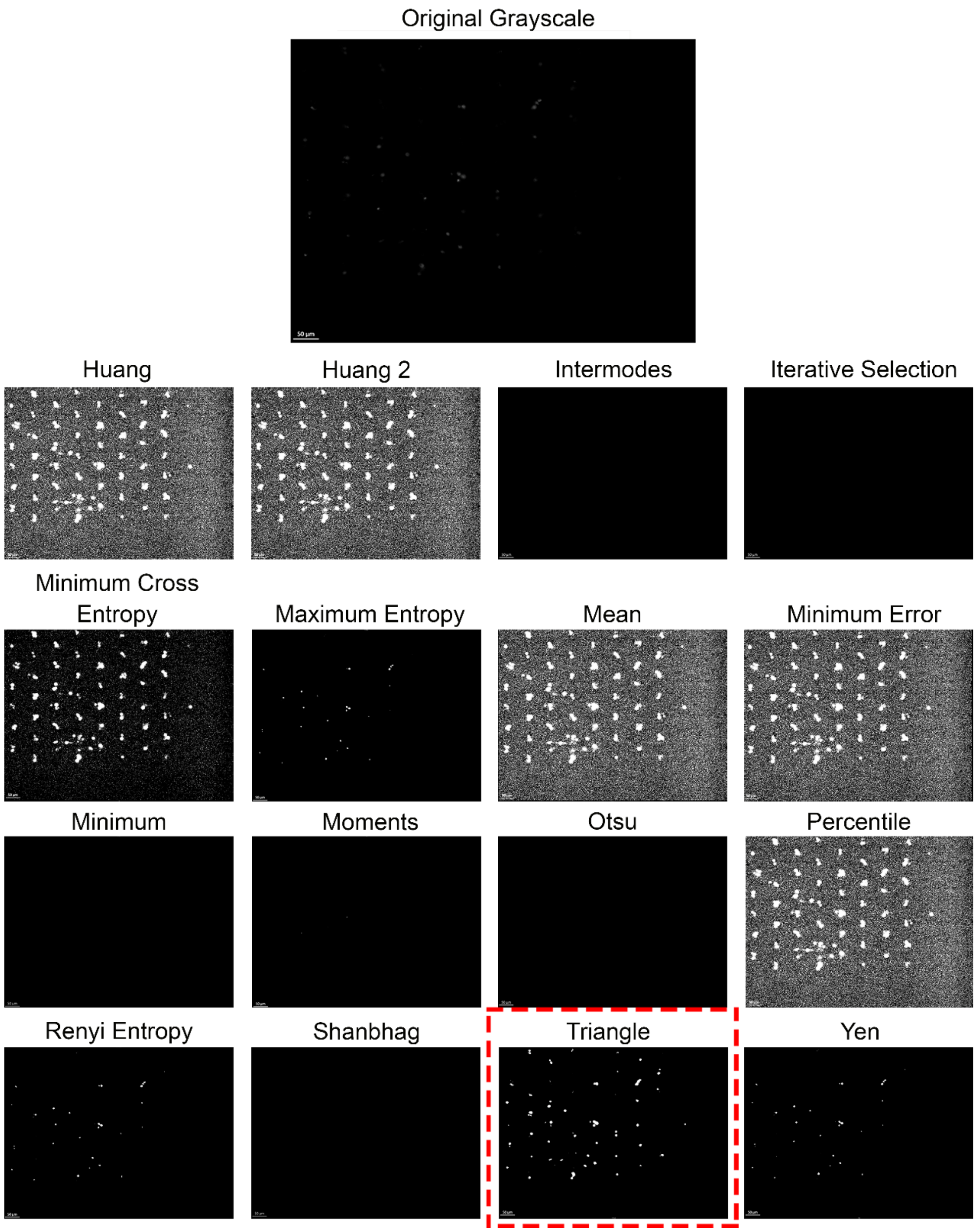

Figure S1. Testing of various thresholding method. Among the 16 types of thresholding methods, the best result was obtained by the Triangle method, and hence, the Triangle method is implemented in ALICE.

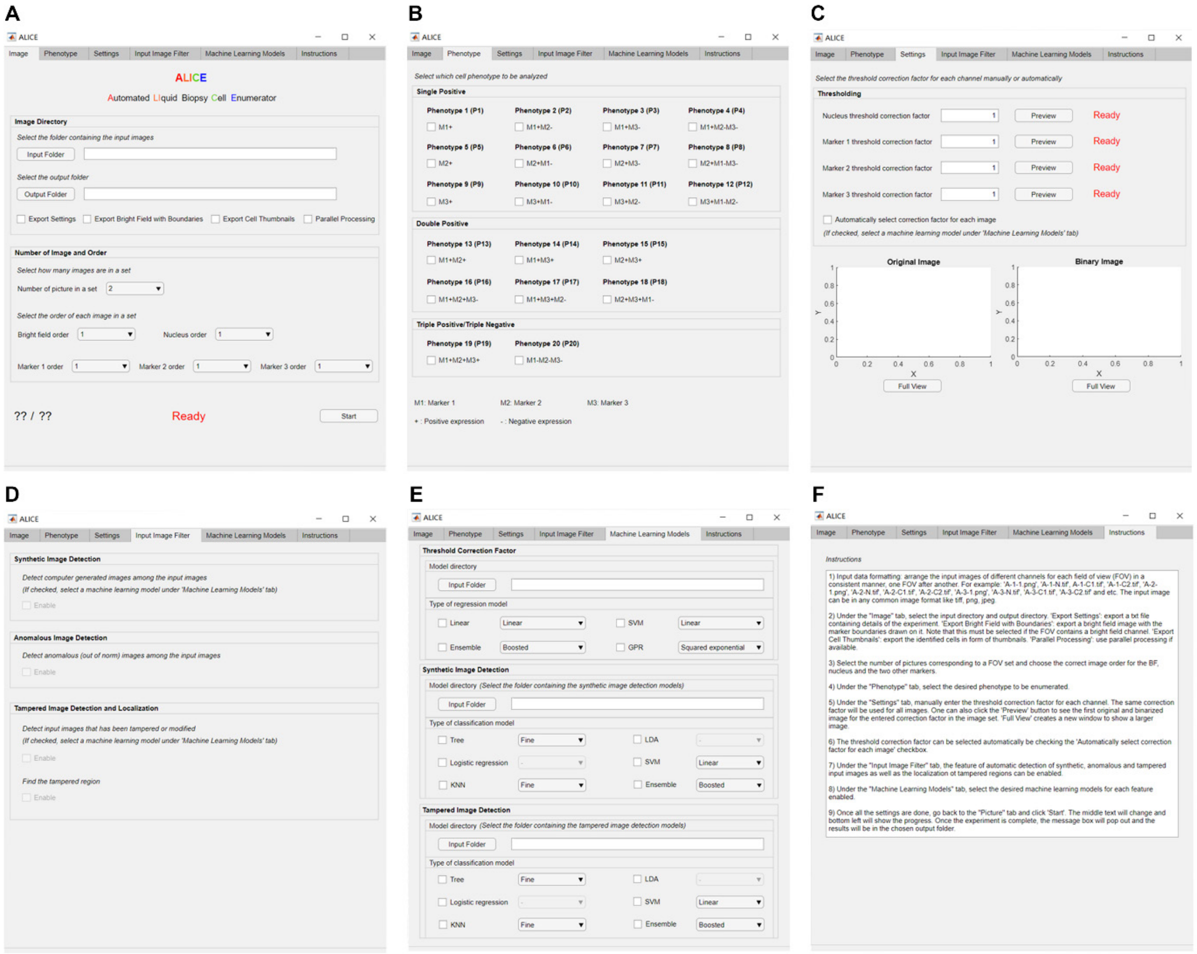

Figure S2. Graphic user interface of ALICE. (A) The interface for choosing the input, output locations and image orders. (B) Selection of desired cell phenotypes to be enumerated by ALICE. (C) Selection of threshold correction factors for each channel. (D) Interface to enable the detection of synthetic, anomalous and tampered input images as well as the localization of the tampered regions. (E) Selection of machine learning models for threshold correction factor determination, synthetic and tampered input images detection. (F) Instructions for using ALICE.

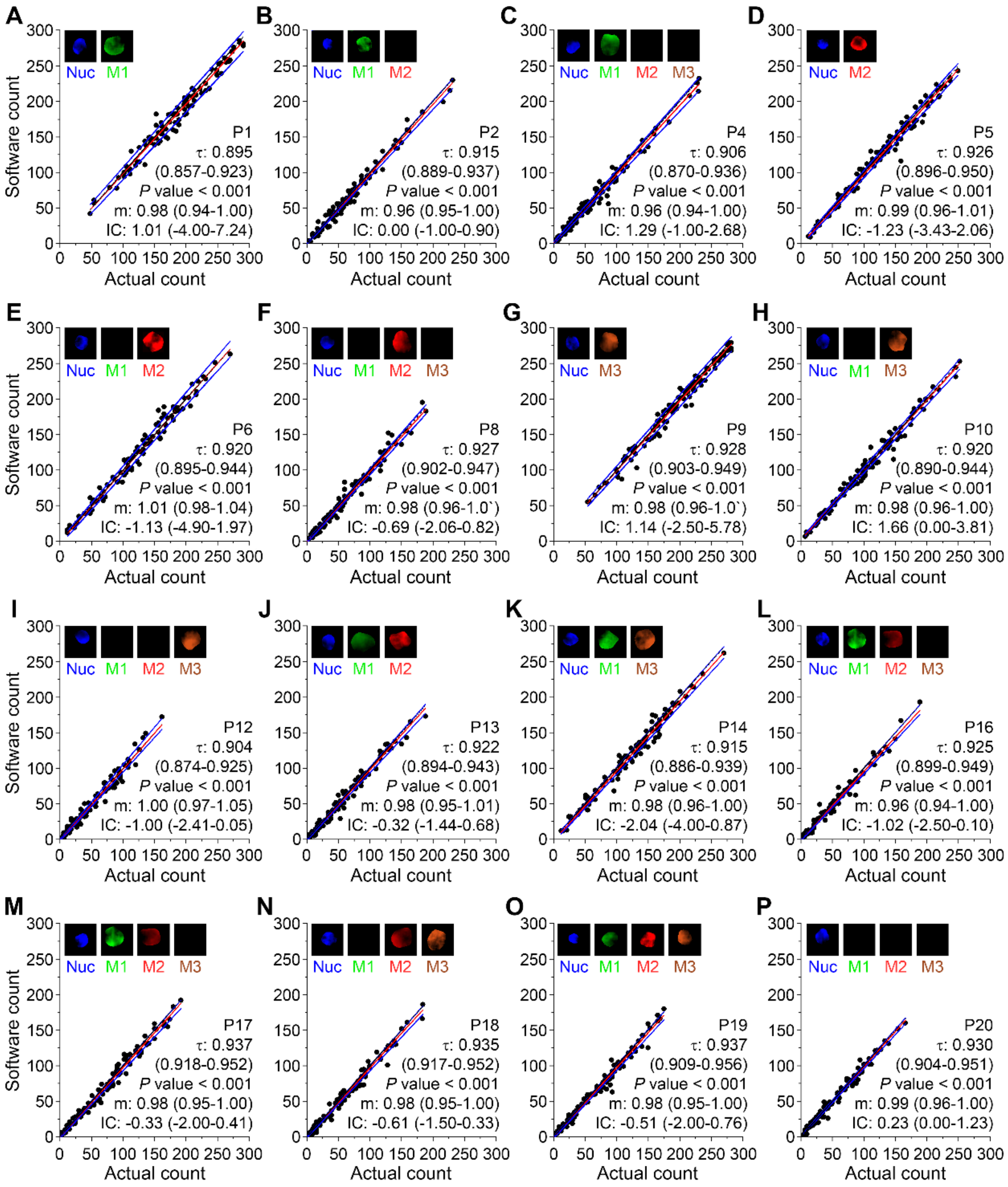

Figure S3. Agreement analysis via Passing-Bablok regression for the remaining 16 phenotypes. The results for (A) P1, (B) P2, (C) P4, (D) P5, (E) P6, (F) P8, (G) P9, (H) P10, (I) P12, (J) P13, (K) P14, (L) P16, (M) P17, (N) P18, (O) P19 and (P) P20 (all *n*=100) are shown whereas the results for P3, P7, P11 and P15 are shown in Figure 3F-I. Black dash lines represent the identity line, red solid lines represent the fitted line and blue solid lines represent the 95% CI of the fitted line. The individual fluorescent channel images are shown as insets. τ denotes the Kendall's correlation coefficient and m denotes the slope. Nuc denotes nucleus, M1 denotes cytoplasm marker 1, M2 denotes cytoplasm marker 2 and M3 denotes cytoplasm marker 3. The definition of the phenotypes is shown in Table S2.

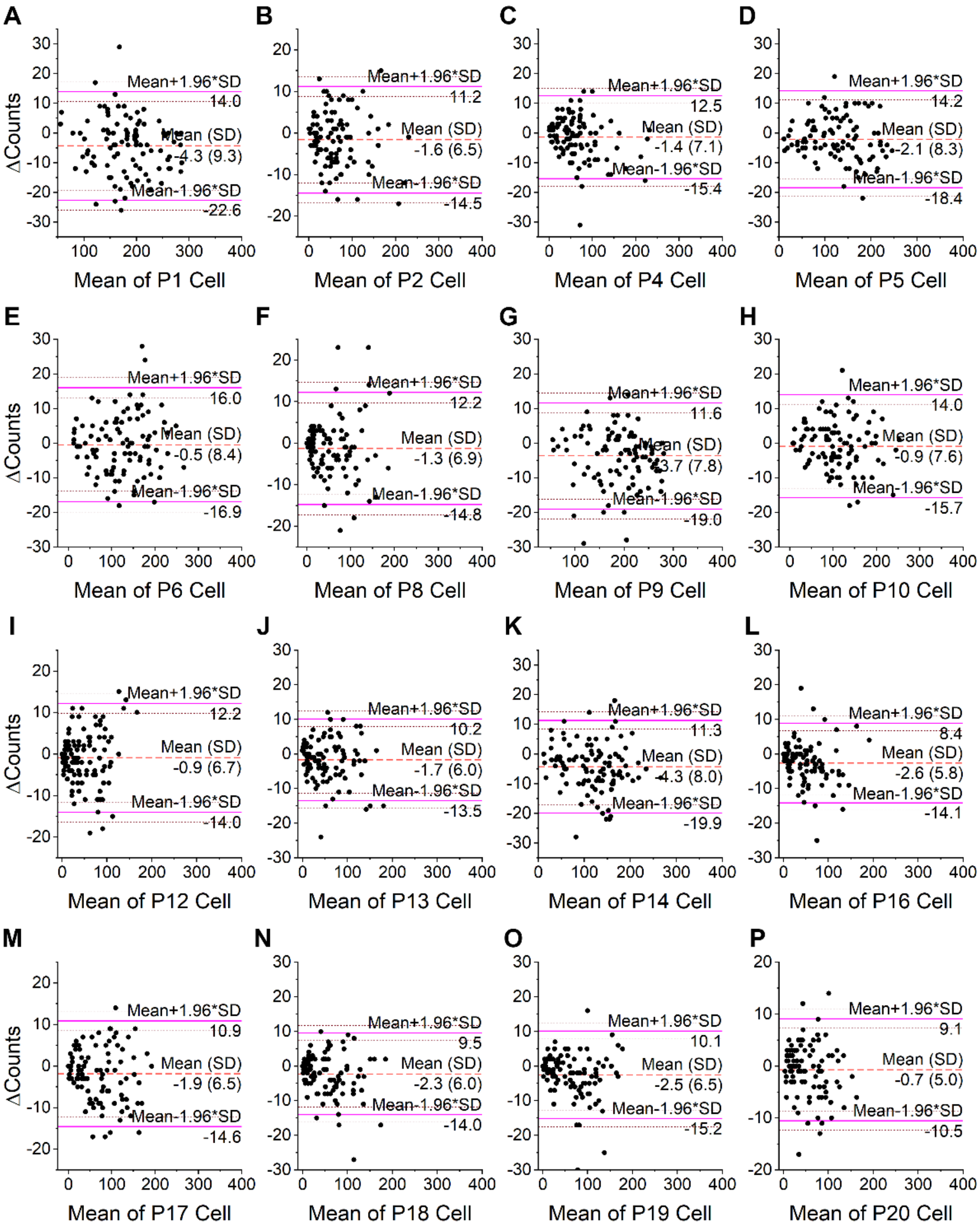

Figure S4. Agreement analysis via Bland-Altman plot for the remaining 16 phenotypes. The results for (A) P1, (B) P2, (C) P4, (D) P5, (E) P6, (F) P8, (G) P9, (H) P10, (I) P12, (J) P13, (K) P14, (L) P16, (M) P17, (N) P18, (O) P19 and (P) P20 (all *n*=100) are shown whereas the results for P3, P7, P11 and P15 are shown in Figure 3J-M. "ΔCount" denotes the difference between the 2 counts. The orange dash lines represent the mean difference between ALICE's count and the simulated ground truth, the purple solid lines represent the 95% limits of agreement and the brown dotted lines represent the 95% CI of the limits of agreements. The definition of the phenotypes is shown in Table S2.

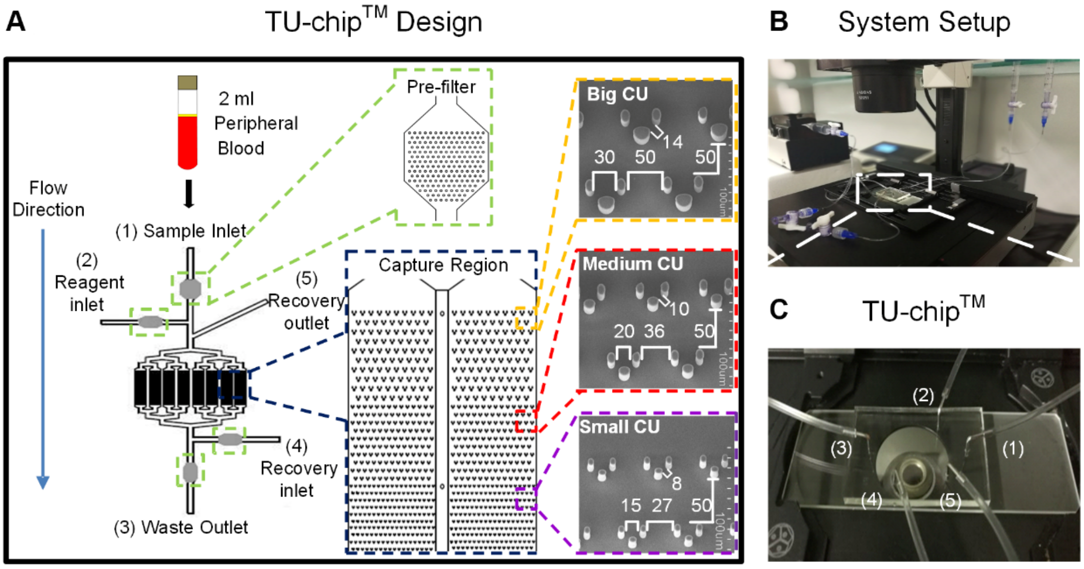

Figure S5. Design of the TU-chip™ and the experimental setup. (A) The design of the TU-chip™ consists of 5 entry-exit points: (1) sample inlet, (2) reagent inlet, (3) waste outlet, (4) recovery inlet and (5) recovery outlet. There are two pre-filter regions to minimize clogging from large particles and a capture region containing capture units (CUs) of three different sizes (big, medium and small) arranged in a spatially gradated manner. (B) System setup for a rapid capturing and identification of CTCs. (C) Enlarged view of the TU-chip™. Adapted with permission from Ref. [1]. Copyright Elsevier, 2019.

Reference

1. Sun Y, Wu G, Cheng KS, Chen A, Neoh KH, Chen S, et al. CTC phenotyping for a preoperative assessment of tumor metastasis and overall survival of pancreatic ductal adenocarcinoma patients. EBioMedicine. 2019; 46: 133-49.

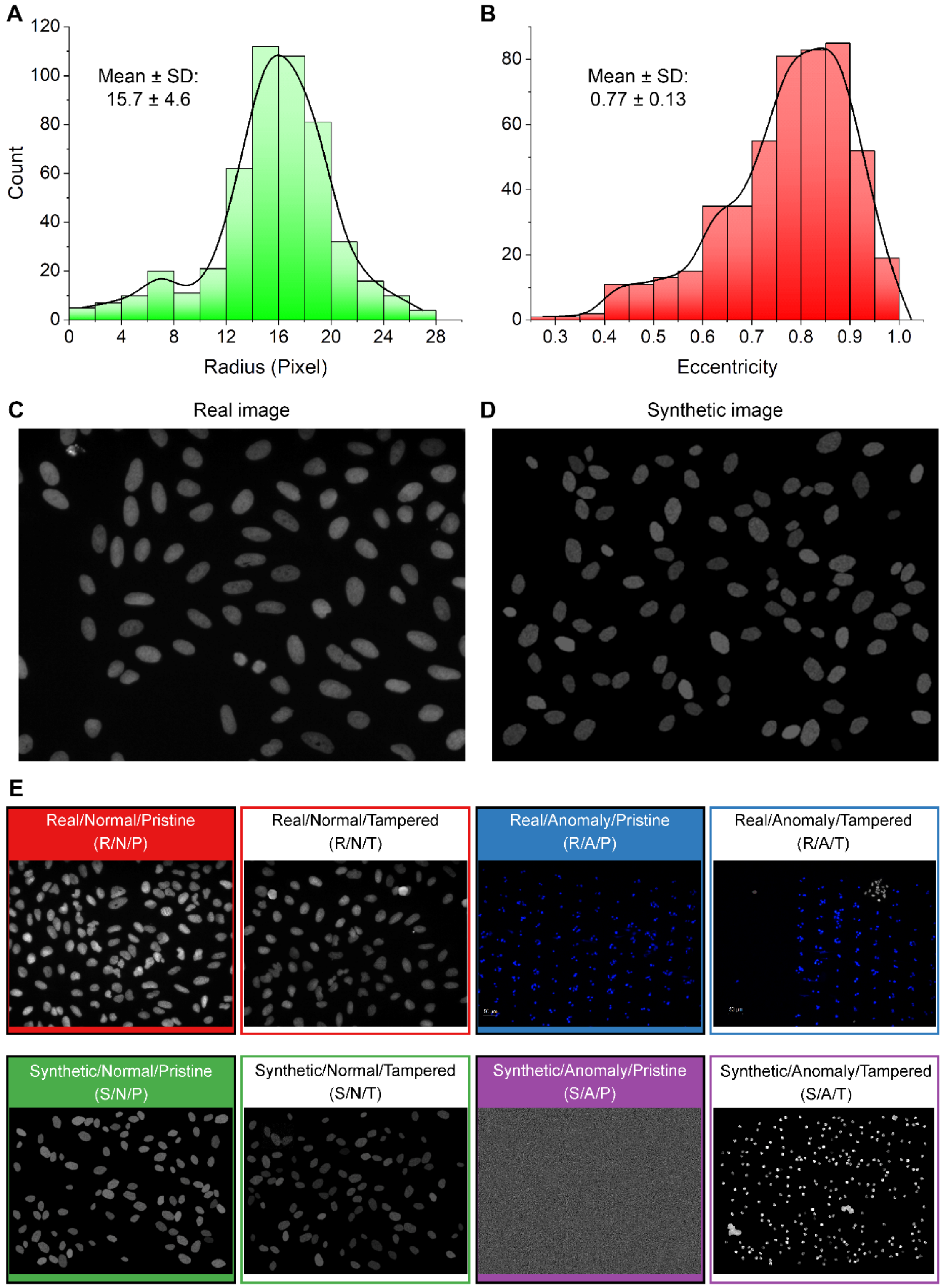

Figure S6. Creation of a realistic synthetic fluorescent image. (A-B) Measurement of the distribution of the radius and eccentricity of 500 cells from the BBBC021v1 image set. (C-D) Example of a real image from the image set and a synthetic image created using SimuCell. (E) Example of each of the 8 possible image types based on whether the input image is either real (R) or synthetic (S), normal (N) or anomalous (A) and pristine (P) or tampered (T).

Supplementary Tables

Table S1. Optimizing machine learning models for an automated selection of threshold correction factors

Model	Term/ Kernel Function	10 × 10-fold CV Set			Test Set			Rank
		RMSE	MAE	Mean	RMSE	MAE	Mean	
Linear	Linear	1.54 (0.97-2.11)	1.00 (0.98-1.02)	1.27	1.39 (1.14-1.46)	1.02 (0.99-1.06)	1.21	9
	Robust	3.32 (1.77-4.87)	1.05 (1.01-1.09)	2.19	1.57 (1.41-1.94)	1.04 (1.00-1.10)	1.31	11
SVM	Linear	1.60 (1.34-1.86)	1.08 (1.07-1.10)	1.34	1.69 (1.57-1.91)	1.13 (1.08-1.18)	1.41	12
	Quadratic	1.60 (0.41-2.80)	0.88 (0.85-0.91)	1.24	1.26 (1.21-1.33)	0.84 (0.81-0.88)	1.05	6
	Cubic	10.83 (2.53-19.14)	3.28 (0.00-6.63)	7.06	4.71 (2.97-8.20)	1.79 (1.67-2.03)	3.25	14
	Fine Gaussian	1.91 (1.90-1.92)	1.18 (1.18-1.19)	1.55	1.58 (1.50-1.68)	0.98 (0.94-1.04)	1.28	10
	Medium Gaussian	1.30 (1.29-1.30)	0.90 (0.90-0.90)	1.10	1.38 (1.32-1.45)	0.93 (0.90-0.98)	1.16	7
	Coarse Gaussian	1.74 (1.74-1.75)	1.22 (1.22-1.23)	1.48	1.83 (1.77-1.92)	1.28 (1.23-1.33)	1.56	13
**Ensemble**	Boosting	1.26 (1.25-1.27)	0.88 (0.88-0.89)	1.07	1.41 (1.34-1.50)	0.96 (0.93-1.01)	1.19	8
	**Random Forest**	**0.88 (0.87-0.89)**	**0.58 (0.57-0.58)**	**0.73**	**0.74 (0.70-0.81)**	**0.50 (0.47-0.52)**	**0.62**	**1**
GPR	Squared Exponential	1.19 (1.18-1.21)	0.83 (0.83-0.84)	1.01	1.18 (1.14-1.24)	0.82 (0.78-0.85)	1.00	5
	Matern 5/2	1.16 (1.15-1.17)	0.81 (0.81-0.81)	0.99	1.15 (1.10-1.19)	0.78 (0.74-0.81)	0.97	4
	Exponential	1.10 (1.09-1.10)	0.74 (0.73-0.74)	0.92	1.06 (1.01-1.11)	0.61 (0.57-0.65)	0.84	2
	Rational Quadratic	1.16 (1.15-1.17)	0.80 (0.79-0.81)	0.98	1.12 (1.08-1.18)	0.75 (0.72-0.78)	0.94	3

CV: cross-validation; RMSE: root mean square error; MAE: mean absolute error; SVM: support vector machine; GPR: Gaussian process regression.

Table S2. Definition of the 20 Phenotypes in ALICE

Phenotype	Definition[a]	Equivalency[b]
P1	M1+	∑ (P2 P3 P4 P13 P14 P16 P17 P19)
P2	M1+ M2-	∑ (P4 P17)
P3	M1+ M3-	∑ (P4 P16)
P4	M1+ M2- M3-	
P5	M2+	∑ (P6 P7 P8 P13 P15 P16 P18 P19)
P6	M2+ M1-	∑ (P8 P18)
P7	M2+ M3-	∑ (P8 P16)
P8	M2+ M1- M3-	
P9	M3+	∑ (P10 P11 P12 P14 P15 P17 P18 P19)
P10	M3+ M1-	∑ (P12 P18)
P11	M3+ M2-	∑ (P12 P17)
P12	M3+ M1- M2-	
P13	M1+ M2+	∑ (P16 P19)
P14	M1+ M3+	∑ (P17 P19)
P15	M2+ M3+	∑ (P18 P19)
P16	M1+ M2+ M3-	
P17	M1+ M3+ M2-	
P18	M2+ M3+ M1-	
P19	M1+ M2+ M3+	
P20	M1- M2- M3-	

M1: marker 1; M2: marker 2; M3: marker 3; [a]: "+" denotes positive expression whereas "-" denotes negative expression; [b]: "∑" denotes sum.

Table S3. Performance of the input image anomaly detection using robust principal component analysis (PCA) with varying combinations of parameters *k* and *α* under 4 different percentage of anomalies

Parameter	Condition (% anomaly)	Precision	Recall	F1 Score	Matthews Correlation Coefficient (MCC)
k = 1, α = 0.50	1%	0.333	1.000	0.500	0.572
	5%	0.833	1.000	0.909	0.908
	10%	0.909	1.000	0.952	0.948
	25%	1.000	1.000	1.000	1.000
k = 1, α = 0.55	1%	0.500	1.000	0.667	0.704
	5%	0.833	1.000	0.909	0.908
	10%	0.909	1.000	0.952	0.948
	25%	1.000	1.000	1.000	1.000
k = 1, α = 0.60	1%	0.500	1.000	0.667	0.704
	5%	0.833	1.000	0.909	0.908
	10%	0.909	1.000	0.952	0.948
	25%	1.000	1.000	1.000	1.000
k = 1, α = 0.65	1%	0.333	1.000	0.500	0.572
	5%	0.833	1.000	0.909	0.908
	10%	0.909	1.000	0.952	0.948
	25%	1.000	1.000	1.000	1.000
**k = 1, α = 0.70**	1%	0.500	1.000	0.667	0.704
	5%	1.000	1.000	1.000	1.000
	10%	1.000	1.000	1.000	1.000
	25%	1.000	1.000	1.000	1.000
k = 1, α = 0.75	1%	1.000	1.000	1.000	1.000
	5%	1.000	1.000	1.000	1.000
	10%	1.000	1.000	1.000	1.000
	25%	-	0.000	0.000	-
k = 2, α = 0.70	1%	0.167	1.000	0.286	0.398
	5%	0.556	1.000	0.714	0.730
	10%	0.714	1.000	0.833	0.826
	25%	0.926	1.000	0.962	0.949
k = 3, α = 0.70	1%	0.083	1.000	0.154	0.272
	5%	0.333	1.000	0.500	0.546
	10%	0.588	1.000	0.741	0.737
	25%	0.893	1.000	0.943	0.926

-: unable to evaluate; *k*: number of principle components to retain; *α*: lower bound of the percentage of uncontaminated observation

Table S4. Optimizing machine learning models for an automated detection of tampered input images

Model	Kernel Function	Tampered Image: 10 × 10-fold CV Set: Sensitivity	Specificity	Accuracy	Mean	Rank	Tampered Image: Test Set: Sensitivity	Specificity	Accuracy	Mean	Rank
Decision Tree	Fine	94.62 (94.39-94.86)	96.99 (96.62-97.35)	95.81 (95.60-96.01)	95.81	3	94.97 (94.34-95.54)	97.70 (97.26-98.10)	96.33 (95.97-96.69)	96.32	4
	Medium	94.36 (94.05-94.67)	93.77 (93.59-93.94)	94.06 (93.91-94.22)	94.06	7	94.67 (93.95-95.34)	93.99 (93.30-94.64)	94.33 (93.83-94.79)	94.33	8
	Coarse	91.36 (91.17-91.54)	91.00 (90.72-91.29)	91.18 (91.09-91.27)	91.18	11	92.16 (91.34-92.83)	90.75 (89.93-91.52)	91.46 (90.88-92.02)	91.46	12
Discriminant Analysis	Linear	89.59 (89.48-89.70)	94.88 (94.81-94.94)	92.23 (92.18-92.29)	92.23	10	90.09 (89.17-90.92)	94.80 (94.11-93.59)	92.45 (91.91-92.98)	92.45	10
Logistic Regression		91.69 (87.02-96.36)	90.31 (85.52-95.09)	91.00 (89.76-92.25)	91.00	13	94.82 (94.16-95.40)	95.55 (94.94-96.11)	95.18 (94.75-95.59)	95.18	7
SVM	Linear	89.34 (89.19-89.50)	93.01 (92.95-93.08)	91.17 (91.10-91.25)	91.17	12	88.96 (87.97-89.76)	93.12 (92.42-93.87)	91.04 (90.43-91.59)	91.04	13
	Quadratic	94.71 (94.60-94.83)	97.66 (97.53-97.80)	96.19 (96.11-96.27)	96.25	2	95.53 (94.86-96.07)	97.88 (97.57-98.36)	96.75 (96.39-97.08)	96.72	3
	Cubic	94.50 (94.36-94.65)	97.02 (96.89-97.15)	95.76 (95.66-95.87)	95.76	4	95.21 (94.59-95.80)	97.19 (96.65-97.58)	96.20 (95.82-96.57)	96.20	5
	Fine Gaussian	94.09 (94.01-94.16)	93.12 (93.04-93.20)	93.60 (93.56-93.65)	93.60	9	94.48 (93.37-95.09)	92.90 (92.20-93.62)	93.69 (93.22-94.24)	93.69	9
	Medium Gaussian	92.71 (92.65-92.78)	97.27 (97.19-97.35)	94.99 (94.94-95.04)	94.99	5	93.29 (92.54-93.99)	98.00 (97.58-98.38)	95.64 (95.22-96.05)	95.64	6
	Coarse Gaussian	82.56 (82.46-82.66)	93.75 (93.68-93.82)	88.15 (88.09-88.21)	88.15	21	82.59 (81.38-83.59)	93.84 (93.08-94.48)	88.22 (87.49-88.81)	88.22	19
KNN	Fine	84.87 (84.71-85.03)	88.77 (88.65-88.89)	86.82 (86.73-86.91)	86.82	22	84.81 (83.68-85.82)	88.17 (87.26-89.05)	86.49 (85.78-87.16)	86.49	20
	Medium	84.68 (86.29-86.67)	96.71 (96.62-96.80)	91.59 (91.49-91.70)	90.99	14	85.25 (84.14-86.19)	98.04 (97.61-98.40)	91.65 (91.05-92.18)	91.65	11
	Coarse	85.24 (85.14-85.34)	95.96 (95.87-96.06)	90.60 (90.56-90.64)	90.60	15	85.06 (84.06-86.11)	96.76 (96.23-97.22)	90.91 (90.29-91.5)	90.91	14
	Cosine	83.98 (83.80-84.15)	96.61 (96.53-96.70)	90.30 (90.21-90.38)	90.30	17	81.74 (80.60-82.81)	**98.25 (97.88-98.60)**	90.00 (89.40-90.58)	90.00	16
	Cubic	84.59 (84.46-84.72)	95.07 (94.97-95.17)	89.83 (89.75-89.91)	89.83	19	82.76 (82.41-83.12)	97.00 (96.84-97.18)	89.88 (89.68-90.09)	89.88	17
	Weighted	86.18 (86.09-86.26)	93.50 (93.37-93.63)	89.84 (89.77-89.90)	89.84	18	86.49 (85.41-87.38)	93.22 (92.48-93.91)	89.86 (89.23-90.48)	89.86	18
**Ensemble**	Boosted	95.69 (95.55-95.82)	97.17 (97.02-97.32)	96.43 (96.37-96.49)	94.43	6	96.46 (95.88-96.93)	97.10 (96.64-97.58)	96.78 (96.39-97.11)	96.78	2
	**Random Forest**	**96.97 (96.89-97.04)**	**98.11 (98.01-98.21)**	**97.54 (97.48-97.60)**	**97.54**	**1**	**97.38 (95.82-97.79)**	97.78 (97.33-98.20)	**97.58 (97.22-97.88)**	**97.58**	**1**
	Subspace Discriminant	86.57 (86.44-86.71)	94.57 (94.50-94.65)	90.57 (90.50-90.65)	90.57	16	87.00 (85.93-87.91)	94.23 (93.59-94.92)	90.61 (89.94-91.15)	90.61	15
	Subspace KNN	86.59 (86.43-86.75)	91.06 (90.80-91.32)	88.82 (88.64-89.00)	88.82	20	79.71 (78.63-80.93)	87.77 (86.80-88.76)	83.74 (83.04-84.52)	83.74	21
	RUSBoosted	94.41 (94.06-94.76)	93.69 (93.34-94.03)	94.05 (93.93-94.16)	94.05	8	94.67 (93.99-95.35)	93.99 (93.29-94.63)	94.33 (93.84-94.78)	94.33	8

CV: cross-validation; SVM: support vector machine; KNN: k-nearest neighbor

Table S5. Optimizing machine learning models for an automated detection of <u>synthetic</u> input images

Model	Kernel Function	Synthetic Image									
		10 × 10-fold CV Set					Test Set				
		Sensitivity	Specificity	Accuracy	Mean	Rank	Sensitivity	Specificity	Accuracy	Mean	Rank
Decision Tree	Fine	99.79 (99.73-99.85)	99.74 (99.62-99.85)	99.77 (99.70-99.83)	99.77	6	99.92 (99.54-100)	99.83 (99.40-100)	99.88 (99.62-99.96)	99.88	3
	Medium	99.84 (99.78-99.90)	99.54 (99.40-99.67)	99.70 (99.64-99.75)	99.69	9	100 (100-100)	99.83 (99.41-100)	99.92 (99.72-100)	99.92	2
	Coarse	86.05 (85.46-86.63)	94.20 (94.15-94.26)	89.86 (89.67-90.25)	90.04	18	83.56 (81.42-85.61)	93.63 (92.22-94.91)	88.39 (87.15-89.64)	88.53	15
Discriminant Analysis	Linear	99.98 (99.91-100)	99.65 (99.63-99.68)	99.83 (99.79-99.86)	99.82	5	**100 (100-100)**	99.75 (99.35-99.92)	99.88 (99.68-99.96)	99.88	3
**Logistic Regression**		99.98 (99.93-100)	**100 (100-100)**	**99.99 (99.96-100)**	**99.99**	**1**	**100 (100-100)**	**100 (100-100)**	**100 (100-100)**	**100**	**1**
**SVM**	Linear	**100 (100-100)**	99.69 (99.62-99.76)	99.85 (99.82-99.88)	99.85	4	**100 (100-100)**	99.66 (99.17-99.92)	99.84 (99.60-99.96)	99.83	4
	**Quadratic**	99.99 (99.95-100)	99.98 (99.97-100)	99.98 (99.94-100)	99.98	2	**100 (100-100)**	**100 (100-100)**	**100 (100-100)**	**100**	**1**
	**Cubic**	99.97 (99.92-100)	99.99 (99.97-100)	99.98 (99.94-100)	99.98	2	**100 (100-100)**	**100 (100-100)**	**100 (100-100)**	**100**	**1**
	Fine Gaussian	97.32 (97.21-97.44)	99.97 (99.95-100)	98.60 (98.54-98.65)	98.63	15	97.38 (96.36-98.13)	**100 (100-100)**	98.63 (98.11-99.04)	98.67	12
	Medium Gaussian	99.89 (99.82-99.96)	99.81 (99.81-99.81)	99.85 (99.82-99.89)	99.85	4	99.85 (99.45-100)	99.75 (99.31-99.92)	99.80 (99.56-99.92)	99.80	5
	Coarse Gaussian	99.97 (99.94-100)	99.31 (99.23-99.40)	99.65 (99.60-99.70)	99.64	10	**100 (100-100)**	99.08 (98.47-99.57)	99.56 (99.28-99.80)	99.55	7
KNN	Fine	99.75 (99.68-99.82)	98.72 (98.61-98.84)	99.26 (99.20-99.32)	99.24	11	99.92 (99.55-100)	98.83 (98.12-99.33)	99.40 (99.04-99.68)	99.38	8
	Medium	99.56 (99.47-99.65)	98.11 (97.95-98.28)	98.87 (98.78-98.96)	98.85	14	99.77 (99.37-99.93)	98.32 (97.46-98.93)	99.08 (98.62-99.40)	99.06	11
	Coarse	99.44 (99.37-99.51)	96.34 (96.24-96.44)	97.96 (97.88-98.03)	97.91	16	99.69 (99.22-99.92)	95.98 (94.84-96.98)	97.91 (97.35-98.39)	97.86	14
	Cosine	99.35 (99.26-99.43)	98.64 (98.51-98.76)	99.01 (98.95-99.06)	99.00	13	99.77 (99.37-99.92)	98.41 (97.47-98.99)	99.12 (98.67-99.44)	99.10	10
	Cubic	98.61 (98.45-98.77)	96.74 (96.52-96.96)	97.71 (97.58-97.84)	97.69	17	98.77 (98.07-99.26)	96.98 (95.98-97.82)	97.91 (97.35-98.39)	97.89	13
	Weighted	99.74 (99.66-99.81)	98.47 (98.29-99.94)	99.13 (99.03-99.23)	99.11	12	99.85 (99.49-100)	98.49 (97.68-99.06)	99.20 (98.78-99.48)	99.18	9
**Ensemble**	Boosted	99.94 (99.89-99.98)	99.82 (99.69-99.94)	99.88 (99.81-99.94)	99.88	3	**100 (100-100)**	99.83 (99.41-100)	99.92 (99.72-100)	99.92	2
	**Random Forest**	99.99 (99.96-100)	99.98 (99.95-100)	**99.99 (99.96-100)**	**99.99**	**1**	**100 (100-100)**	**100 (100-100)**	**100 (100-100)**	**100**	**1**
	Subspace Discriminant	99.97 (99.91-100)	99.56 (99.53-99.58)	99.77 (99.74-99.80)	99.77	6	**100 (100-100)**	99.75 (99.32-99.92)	99.88 (99.68-99.96)	99.88	3
	Subspace KNN	99.83 (99.83-99.90)	99.62 (99.57-99.67)	99.75 (99.72-99.78)	99.73	8	**100 (100-100)**	99.41 (98.85-99.75)	99.72 (99.44-99.88)	99.71	6
	RUSBoosted	99.92 (99.83-100)	99.56 (99.41-99.72)	99.75 (99.66-99.84)	99.74	7	**100 (100-100)**	99.83 (99.41-100)	99.92 (99.72-100)	99.92	2

CV: cross-validation; SVM: support vector machine; KNN: k-nearest neighbor

Table S6. Comparison of classifiers in detecting synthetic images in the final testing dataset

Model	Condition	Recall	Precision	F1 Score	Matthews Correlation Coefficient (MCC)
Random forest	S/A/P	0.000	-	0.000	-
	S/N/P	0.000	0.000	0.000	-0.025
	R/A/P	1.000	0.375	0.546	0.606
	R/N/P	0.928	0.984	0.955	0.616
	S/A/T	1.000	1.000	1.000	1.000
	S/N/T	0.000	0.000	0.000	-0.007
	R/A/T	0.583	0.875	0.700	0.712
	R/N/T	0.417	0.167	0.238	0.250
Logistic regression	S/A/P	1.000	1.000	1.000	1.000
	S/N/P	0.000	0.000	0.000	-0.015
	R/A/P	1.000	0.600	0.750	0.772
	R/N/P	0.964	0.984	0.974	0.728
	S/A/T	1.000	1.000	1.000	1.000
	S/N/T	0.000	0.000	0.000	-0.004
	R/A/T	0.583	0.875	0.700	0.718
	R/N/T	0.583	0.212	0.311	0.340
Quadratic SVM	S/A/P	0.000	0.000	0.000	1.000
	S/N/P	0.833	0.714	0.769	0.624
	R/A/P	1.000	0.387	0.558	0.772
	R/N/P	0.981	0.999	0.990	0.863
	S/A/T	1.000	1.000	1.000	1.000
	S/N/T	0.500	0.750	0.600	0.747
	R/A/T	0.583	0.875	0.700	0.712
	R/N/T	0.667	0.307	0.421	0.422
**Cubic SVM**	S/A/P	1.000	1.000	1.000	1.000
	S/N/P	0.833	0.476	0.606	0.769
	R/A/P	1.000	0.600	0.750	0.616
	R/N/P	0.974	0.999	0.986	0.897
	S/A/T	1.000	1.000	1.000	1.000
	S/N/T	0.750	0.750	0.750	0.609
	R/A/T	0.583	0.875	0.700	0.712
	R/N/T	0.583	0.318	0.412	0.444

SVM: support vector machine; R: real; S: synthetic; N: normal; A: anomalous; P: pristine; T: tampered; -: unable to evaluate due to absence of predicted positive cases

Table S7. Regression analysis of the counts of five different circulating tumor cell (CTC) phenotypes obtained from ALICE (Automated Liquid Biopsy Cell Enumerator). Zero-inflated models have a zero part and a count part whereas nonzero-inflated models only have a count part.

Variable	Factor	Zero Part					Count Part				
		Estimate	SE	OR	OR 95% CI	*P* value	Estimate	SE	OR	OR 95% CI	*P* value
HE4- CTC	Method										
(ZIP)	ALICE	-0.758	0.754	0.469	0.107-0.720	0.315	0.343	0.340	1.409	0.723-2.744	0.314
	Manual	Ref.					Ref.				
	Intercept	-0.112	0.546	0.894	0.306-2.607	0.838	-0.444	0.288	0.641	0.365-1.128	0.123
HE4+ CTC	Method										
(P)	ALICE						-0.956	0.526	0.385	0.137-1.079	0.069
	Manual						Ref.				
	Intercept						-2.255	0.277	0.105	0.061- 0.181	<0.001
E-CTC	Method										
(ZIP)	ALICE	-0.081	1.062	0.922	0.115-7.393	0.939	-1.600	0.996	0.202	0.029-1.422	0.108
	Manual	Ref.					Ref.				
	Intercept	2.322	0.358	10.196	5.055-20.567	< 0.001	0.833	0.242	2.300	1.431-3.696	< 0.001
M-CTC	Method										
(P)	ALICE	1.092	0.755	2.980	0.679-13.089	0.148	0.540	0.668	1.716	0.463-6.355	0.418
	Manual	Ref.					Ref.				
	Intercept	1.361	0.556	3.900	1.312-2.451	0.014	-0.422	0.477	0.656	0.257-1.670	0.377
H-CTC	Method										
(ZIP)	ALICE						0.329	0.204	1.389	0.931-2.073	0.108
	Manual						Ref.				
	Intercept						-0.442	0.151	0.643	0.478-0.864	0.003

SE: standard error; OR: odds ratio; 95% CI: 95% confidence interval; HE4: human epididymis secretory protein 4; CTC: circulating tumor cell; *E*-CTC: epithelial CTC; *M*-CTC: mesenchymal CTC; *H*-CTC: hybrid CTC; P: Poisson; ZIP: zero-inflated Poisson; Ref.: reference

Table S8. Correlation matrix of circulating hybrid cells (CHCs) and circulating tumor cells (CTCs) (Spearman's *ρ* and *P* value in parenthesis)

	E-CTC	*M*-CTC	*H*-CTC	*T*-CTC
CHC-1	0.011 (0.952)	-0.064 (0.726)	-0.096 (0.602)	-0.040 (0.828)
CHC-2	0.125 (0.495)	-0.086 (0.639)	-0.039 (0.876)	-0.029 (0.876)
CHC-T	0.063 (0.731)	-0.133 (0.467)	-0.101 (0.581)	-0.073 (0.693)

CTC: circulating tumor cell; E-CTC: epithelial CTC; M-CTC: mesenchymal CTC; H-CTC: hybrid CTC; T-CTC: total CTC; CHC: circulating hybrid cell; CHC-T: CHC-Total

Table S9. Diagnostic performance of circulating hybrid cell (CHC)-1 and CHC-Total (CHC-T) in differentiating pancreatic ductal adenocarcinoma (PDAC) patients with lymph node metastasis in the training dataset

Variable	Cutoff (cells/2 ml)	Youden's index	Sensitivity	Specificity	PPV	NPV	Accuracy
CHC-1	1	0.615	0.615 (0.369-0.898)	1.000 (1.000-1.000)	1.000 (1.000-1.000)	0.689 (0.486-0.914)	0.792 (0.667-0.958)
CHC-T	1	0.794	0.770 (0.605-1.000)	0.818 (0.636-1.000)	0.833 (0.667-1.000)	0.750 (0.708-1.000)	0.792 (0.708-1.000)

PPV: positive predictive value; NPV: negative predictive value; CHC: circulating hybrid cell; CHC-T: CHC-Total
